# Hydrogen peroxide-assisted oxidative desulfurization: mechanisms and catalytic perspectives

**DOI:** 10.3389/fchem.2026.1861354

**Published:** 2026-07-23

**Authors:** Ali Ali-zade, Elmir Babayev, Irina Tarkhanova, Igor Nasirov, Dong Zhiguo

**Affiliations:** 1 Azerbaijan State Oil and Industry University, Baku, Azerbaijan; 2 Institute of Catalysis and Inorganic Chemistry named after M. Nagiyev, Baku, Azerbaijan; 3 Department of Chemistry, Lomonosov Moscow State University, Moscow, Russia; 4 Energy Research Institute, Qilu University of Technology, Jinan, China

**Keywords:** catalysis, hydrogen peroxide (H2O2), oxidative desulfurization (ODS), reaction mechanisms, reactive oxygen species (ROS), sulfur removal

## Abstract

The growing environmental concerns and stricter global fuel regulations on sulfur content have accelerated the search for efficient desulfurization technologies. Oxidative Desulfurization (ODS) has emerged as a promising complement or alternative to conventional Hydrodesulfurization (HDS), offering advantages such as mild operating conditions, reduced energy consumption, and superior performance in removing refractory sulfur compounds. Among various oxidants, hydrogen peroxide (H_2_O_2_) has attracted significant interest due to its environmental compatibility, cost-effectiveness, and the benign nature of its by-products—water and oxygen. This review emphasizes mechanistic insights into H_2_O_2_-based ODS, focusing on the activation of H_2_O_2_ and subsequent oxidation of sulfur species. Catalytic systems such as organic acids, polyoxometalates (POMs), ionic liquids (ILs), deep eutectic solvents (DESs), transition metal oxides, and metal-organic frameworks (MOFs) have been widely explored for enhancing H_2_O_2_ activation. The generation of reactive oxygen species (ROS), especially hydroxyl radicals (•OH), plays a pivotal role in oxidizing organic sulfur compounds into polar sulfoxides and sulfones, enabling their easy separation. Recent progress in catalyst design has yielded highly efficient and reusable systems under mild conditions, underscoring the potential of H_2_O_2_-assisted ODS as a sustainable pathway for producing ultra-low sulfur fuels.

## Graphical Abstract

**Figure:**
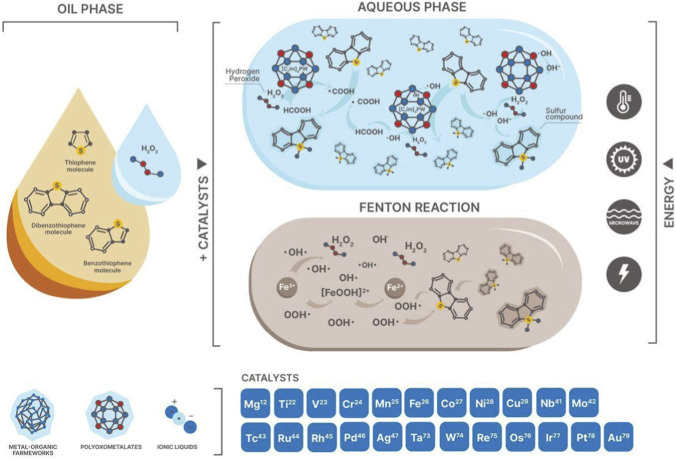

## 1 Background

The presence of sulfur-containing compounds in fossil fuels remains one of the key environmental and technological challenges in the energy sector (Ng and Choi, 2025; Wu et al., 2024a; Yu et al., 2024). During combustion, these compounds are converted into sulfur oxides (SO_x_), which contribute to acid rain formation, atmospheric pollution, and adverse effects on human health. Global regulations (e.g., IMO 2020 for marine fuels, Euro VI/7 for automotive fuels, and ultra-low sulfur diesel standards) mandate a drastic reduction of sulfur content in fuels (often <10–15 ppm) (Zaidi et al., 2023; Wu et al., 2023; Li et al., 2023; Rezaee et al., 2021).

Meeting these stringent requirements necessitates the development and implementation of highly effective desulfurization technologies (Haruna et al., 2022).

**FIGURE 1:**
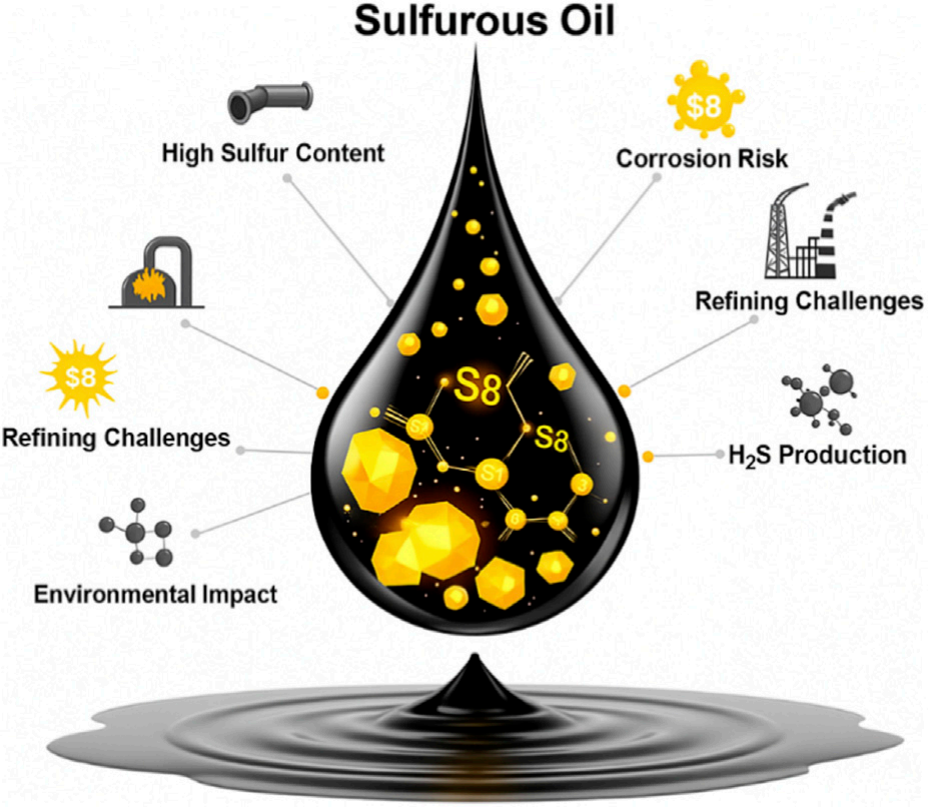
Schematic representation of the challenges associated with sulfurous oil

To meet these requirements, efficient desulfurization technologies are essential. Consequently, regulatory standards on sulfur content in motor fuels are being tightened worldwide, which in turn stimulates the development of more effective desulfurization methods (Figure 1) (Campos-Martín et al., 2010; Xu et al., 2024a).

### 1.1 Modern desulfurization processes

**FIGURE 2:**
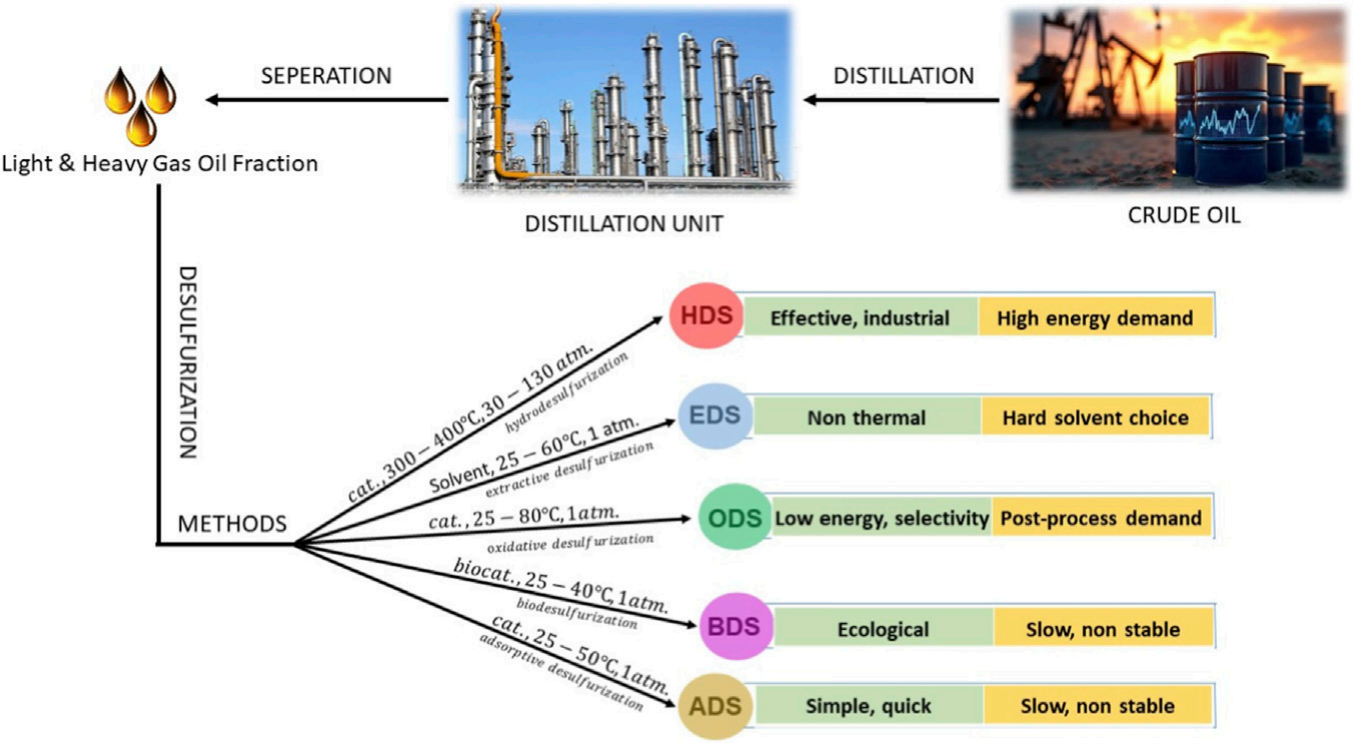
Desulfurization process flow for raw materials.

Modern desulfurization processes represent advanced technologies employed for the removal of sulfur compounds from petroleum fractions and gaseous products. The primary objectives of these processes are to improve fuel quality, mitigate environmental pollution, and prevent catalyst poisoning (Figure 2).

Conventional desulfurization processes, such as hydrodesulfurization (HDS), have been the industry standard for decades. HDS involves reacting sulfur compounds with hydrogen over a catalyst at high temperatures (typically 300 °C–400 °C) and pressures (20–60 bar) to produce hydrogen sulfide (H_2_S) gas. While effective for readily removable thiols (Dedual et al., 2014), sulfides (Zhang et al., 2006), and disulfides (Jeevanandam et al., 2005), HDS faces significant challenges when it comes to “refractory” sulfur compounds, such as dibenzothiophene (DBT) (Trongkaew et al., 2011) and its alkylated derivatives (e.g., 4,6-dimethyldibenzothiophene, 4,6-DMDBT) (Wu et al., 2024b). The resonance stabilization and steric hindrance of these compounds, attributed to π-electron delocalization within aromatic rings, make them exceptionally resistant to HDS, often requiring harsher conditions, increased hydrogen consumption, and higher operational costs.

Consequently, there is an urgent need for alternative or complementary desulfurization methods that can operate under milder conditions, reduce hydrogen consumption, and effectively target these refractory sulfur species.

### 1.2 Oxidative desulfurization (ODS) method

Against this background, the oxidative desulfurization (ODS) method has attracted increasing attention as a promising alternative to hydrodesulfurization (HDS) (Azeez and Ganiyu, 2023; Boldrin et al., 2016; Nazari et al., 2017; Zhang et al., 2025). ODS proceeds under mild conditions, provides high selectivity toward refractory sulfur-containing compounds, and can be integrated into existing fuel purification schemes, including as a post-treatment step following HDS (Mohebali and Ball, 2016; Paiva et al., 2014). The essence of the method lies in the catalytic oxidation of sulfur compounds to the corresponding sulfones or sulfoxides, followed by their removal through extraction or adsorption. Both homogeneous (Rajendran et al., 2020; Thoda et al., 2023; Zhao et al., 2025) and heterogeneous catalysts (Ahmed et al., 2025; Ebrahimi and Bandehali, 2025; Ng et al., 2024) have been investigated for ODS, in combination with various oxidants (hydrogen peroxide, organic peroxides, ozone, oxygen, etc.) (Liu et al., 2020; Ma et al., 2014; Ma et al., 2015; Shi et al., 2016; Yuan et al., 2022). Typically, ODS operates at lower temperatures (often <100 °C) and under atmospheric or low pressure compared to HDS. In addition, it does not require hydrogen, thus eliminating the need for costly H_2_ infrastructure.

### 1.3 Fenton-like mechanism

One of the key challenges in the development of ODS technology remains the diversity and complexity of oxidation mechanisms, which depend on the nature of the catalyst, oxidant, and reaction medium. The most extensively studied pathways include the Fenton mechanism (Firak et al., 2025; Messele et al., 2019; Zhao et al., 2022), based on the generation of highly reactive •OH radicals in the presence of Fe^2+^ and hydrogen peroxide. This mechanism is characterized by high activity (Meyerstein, 2021), but low selectivity (Armandsefat et al., 2024), which limits its application for the selective oxidation of organosulfur compounds in fuels.

### 1.4 Peroxo complexes

Another important direction involves processes mediated by peroxo complexes of transition metals such as Mo, W, V, or Ti (Anisimov et al., 2003; Jiang et al., 2020a; Torres-García et al., 2011). In these cases, the formation of active intermediates occurs via oxygen transfer, and the mechanism may involve either one-electron or two-electron steps. This pathway provides greater controllability and selectivity, particularly in homogeneous systems. These oxygen-transfer events can be made explicit through balanced equations: (Tanimu et al., 2022; Vin et al., 2008) (Equations 1–4):

$${\text{MoO}}_{4}^{2-}+2H_{2}O_{2}+2H^{+}\to \left[\text{MoO}{\left(O_{2}\right)}_{2}\left(H_{2}O\right)\right]+2H_{2}O$$ (1)

$$\left[\text{MoO}{\left(O_{2}\right)}_{2}\left(H_{2}O\right)\right]+\text{DBT}\to \left[{\text{MoO}}_{2}{\left(O_{2}\right)}_{2}\left(H_{2}O\right)\right]+\text{DBTO}$$ (2)

$$\left[{\text{MoO}}_{2}{\left(O_{2}\right)}_{2}\left(H_{2}O\right)\right]+H_{2}O_{2}\to \left[\text{MoO}{\left(O_{2}\right)}_{2}\left(H_{2}O\right)\right]+H_{2}O$$ (3)

$$\left[\text{MoO}{\left(O_{2}\right)}_{2}\left(H_{2}O\right)\right]+\text{DBTO}\to \left[{\text{MoO}}_{2}{\left(O_{2}\right)}_{2}\left(H_{2}O\right)\right]+{\text{DBTO}}_{2}$$ (4)

Tungsten (VI) systems follow an analogous mechanism but typically involve assembly of a higher-nuclearity species in the presence of phosphate, namely, the Venturello–Ishii anion {PO_4_ [WO(O_2_)_2_]_4_}^3-^, which is the active oxygen-transfer agent in many heteropolyacid-based ODS catalysts (Equations 5–7):

$${\text{WO}}_{4}^{2-}+2H_{2}O_{2}+H^{+}\to {\left[\text{WO}{\left(O_{2}\right)}_{2}\left(\text{OH}\right)\right]}^{-}+2H_{2}O$$ (5)

$${\text{PO}}_{4}^{3-}+4{\left[\text{WO}{\left(O_{2}\right)}_{2}\left(\text{OH}\right)\right]}^{-}\to {\left\{{\text{PO}}_{4}{\left[\text{WO}{\left(O_{2}\right)}_{2}\right]}_{4}\right\}}^{3-}+4{\text{OH}}^{-}$$ (6)

$${\left\{{\text{PO}}_{4}{\left[\text{WO}{\left(O_{2}\right)}_{2}\right]}_{4}\right\}}^{3-}+\text{DBT}\to \left(\text{reducedPOM}\right)+\text{DBTO}$$ (7)

In all cases the substrate transformation chain DBT → DBTO → DBTO_2_ proceeds through the rate-determining first oxygen-transfer step, and the net stoichiometry is identical (DBT +2 H_2_O_2_ → DBTO_2_ + 2 H_2_O), with the metal centre acting catalytically. This electrophilic two-electron oxygen-transfer mechanism, which avoids free-radical intermediates, is the principal reason for the high selectivity of peroxo-complex-based ODS systems toward sulfoxide and sulfone formation, and accounts for the long-standing dominance of Mo- and W-based catalysts (POMs, peroxomolybdates, the Venturello–Ishii anion) in industrial-style ODS systems.

### 1.5 Heterogeneous radical oxidation

Equally intriguing are mechanisms realized under conditions of heterogeneous radical oxidation, where reactive oxygen species (O_2_•^-^, ^1^O_2_, •OOH) are generated on the surface of solid catalysts—typically metal oxides with oxygen vacancies (Piscopo et al., 2020). In such systems, the acid–base properties and structural features of the catalyst surface play a crucial role.

A representative example of such a heterogeneous radical pathway is the aerobic oxidative desulfurization of dibenzothiophene (DBT) over V–Nb mixed oxides containing surface oxygen vacancies (V_O_), in which molecular oxygen is activated through a multistep cycle involving reduced V^4+^/Nb^4+^ centres and superoxide radicals (•O_2_^−^) as the dominant reactive oxygen species (Piscopo et al., 2020). The mechanism can be summarized by the following stoichiometric steps:

*Generation of surface oxygen vacancies* (thermal activation of the catalyst under inert atmosphere):

$$2{\left[V^{5+}–O–{\text{Nb}}^{5+}\right]}_{\text{surf}}\to 2{\left[V^{4+}–V_{O}–{\text{Nb}}^{4+}\right]}_{\text{surf}}+O_{2}↑$$

*Chemisorption and activation of molecular oxygen at the oxygen vacancy*, forming a surface peroxo/superoxo intermediate; the bridging V_O_ site donates an electron to the adsorbed O_2_, generating a surface-bound superoxide radical:

$${\left[V^{4+}–V_{O}–{\text{Nb}}^{4+}\right]}_{\text{surf}}+O_{2}\to {\left[V^{5+}–\left(•O_{2}^{–}\right)–{\text{Nb}}^{5+}\right]}_{\text{surf}}$$

*First oxygen transfer to DBT*—the surface superoxide species attacks the sulfur atom (high *f* ^0^(*r*) Fukui index, 0.213), producing the sulfoxide (DBTO) and a surface-bound mono-oxo species:

$${\left[V^{5+}–\left(•O_{2}^{–}\right)–{\text{Nb}}^{5+}\right]}_{\text{surf}}+C_{12}H_{8}S\left(\text{DBT}\right)\to {\left[V^{5+}–O–{\text{Nb}}^{5+}\right]}_{\text{surf}}+C_{12}H_{8}\text{SO}\left(\text{DBTO}\right)$$

*Second oxygen transfer and regeneration of the active site*—a second activated oxygen species oxidizes DBTO to the corresponding sulfone (DBTO_2_), restoring the reduced V^4+^/Nb^4+^ pair and the oxygen vacancy that initiates the next catalytic cycle:

$$C_{12}H_{8}\text{SO}+{\left[V^{5+}–\left(•O_{2}^{–}\right)–{\text{Nb}}^{5+}\right]}_{\text{surf}}\to C_{12}H_{8}{\text{SO}}_{2}\left({\text{DBTO}}_{2}\right)+{\left[V^{4+}–V_{O}–{\text{Nb}}^{4+}\right]}_{\text{surf}}$$

$$C_{12}H_{8}S+O_{2}\to C_{12}H_{8}{\text{SO}}_{2}\left(\text{catalyst}:V–\text{Nb mixed oxide}/V_{O}\right)$$

The role of the oxygen vacancies is thus two-fold: they act as adsorption and electron-donor sites that activate molecular O_2_ into superoxide radicals (•O_2_^−^), and they govern the redox cycling of V^5+^/V^4+^ and Nb^5+^/Nb^4+^ pairs that drive the sequential oxidation DBT → DBTO → DBTO_2_. The dominance of •O_2_^−^ over •OH in this type of heterogeneous radical mechanism has been confirmed by EPR spin-trapping (DMPO–•O_2_^−^ adduct) and by selective radical-scavenging experiments showing that *p*-benzoquinone (a superoxide scavenger) strongly suppresses the catalytic activity, while *tert*-butanol (a hydroxyl-radical scavenger) does not.

### 1.6 Peroxyacids and organic peroxides

It is also worth noting the less explored but promising ionic oxidation pathways involving peroxyacids or organic peroxides (Maslova et al., 2021; Ullah et al., 2022), where the reaction may proceed through the formation of associates between the organosulfur substrate, the oxidant, and the catalyst, bypassing free-radical stages.

The absence of a universal mechanistic description highlights the need for a systematic comparison of pathways using both experimental and computational approaches. Such efforts will not only clarify the differences in activity among known systems but also enable the rational design of new catalysts with tailored selectivity.

This review provides a critical analysis of the mechanistic foundations of oxidative desulfurization (ODS), with emphasis on both homogeneous and heterogeneous catalytic systems. Particular attention is devoted to reaction mechanisms, catalyst design strategies, and the influence of substrate molecular structure and reaction medium parameters on process efficiency. Key achievements in the ODS field are discussed, along with existing limitations and challenges, as well as prospects for the development of environmentally benign and industrially viable desulfurization technologies based on modern approaches.

On the basis of this analysis, it is concluded that a systematic classification of ODS reaction mechanisms and the integration of mechanistic studies with catalyst design are essential. This will allow the transition from an empirical selection of reaction conditions to the rational design of efficient and sustainable processes.

## 2 Overview of oxidative desulfurization (ODS)

### 2.1 Fundamental principle of ODS

The ODS process fundamentally involves two main stages. The first stage is the oxidation of sulfur compounds. Organic sulfur species, such as thiophenes, benzothiophenes (BT), dibenzothiophenes (DBT), and their alkylated derivatives, are oxidized into more polar species, namely, sulfoxides and sulfones (Choi et al., 2021). This conversion occurs by adding oxygen atoms to the sulfur without breaking the carbon-sulfur bonds. Sulfoxides are typically intermediate products, while sulfones are the final, more stable oxidized forms achieved under appropriate conditions, such as excess oxidant. The polarity of these oxidized products is significantly higher than their original sulfur-containing forms, facilitating their subsequent separation.

The second stage involves the separation of the oxidized products. The polar sulfoxides and sulfones are separated from the non-polar fuel matrix. Common separation techniques include liquid-liquid extraction (LLE) using polar solvents such as acetonitrile (MeCN) (Collins et al., 1997), adsorption onto suitable adsorbents, or precipitation. This separation results in a low-sulfur “green fuel.”

The downstream fate of the oxidized sulfur compounds (sulfoxides and sulfones) extracted from the fuel matrix is a critical, yet frequently overlooked, aspect of the overall ODS process economy and environmental footprint. Because sulfones retain the hydrocarbon backbone of the parent thiophenic substrates (e.g., DBTO_2_ contains the entire biphenyl skeleton of DBT), simple disposal as waste leads to unacceptable yield loss of valuable hydrocarbons and generates a sulfur-rich solid residue that requires special handling. Several strategies have therefore been developed to manage, recover, or valorize this sulfone-rich stream.

First, sulfones can be cracked over solid base catalysts (alkali or alkaline-earth hydroxides, carbonates, or mixed metal oxides) at moderate temperatures (200 °C–380 °C, atmospheric pressure) to cleave the C–S bond and release SO_x together with the parent hydrocarbon or its phenolic derivative, which can be returned to the fuel pool. This approach significantly reduces yield loss compared with simple landfill disposal and integrates well with existing refinery configurations (Koseoglu, 2013; Koseoglu and Bourane, 2013). The integrated sulfone decomposition route, in which the ODS reactor is directly coupled with a downstream cracking unit, has been particularly emphasized as a way to eliminate the need for a separate sulfone-separation step and to recover the hydrocarbon portion of the sulfone molecule for return to the fuel pool (Koseoglu and Bourane, 2013).

Second, the SO_x/H_2_S released during sulfone decomposition can be routed to the existing Claus sulfur recovery unit and converted into elemental sulfur—a commodity feedstock for sulfuric acid production, fertilizers, and the rubber industry—closing the sulfur balance of the refinery without the need for dedicated waste-treatment infrastructure.

Third, beyond direct destruction of the sulfone, the principal product of DBT oxidation—dibenzothiophene-S,S-dioxide (DBTSO_2_) — is in fact a privileged building block in organic materials chemistry, owing to its rigid, planar, thermally stable scaffold and strong electron-accepting character. DBTSO_2_-based derivatives have been widely exploited as deep-blue emitters and host materials in organic light-emitting diodes (OLEDs), as photocatalysts for visible-light-driven H_2_ evolution, as donor–acceptor units in solar-cell active layers, and as rigid struts in microporous polymer networks. While most current synthetic routes prepare DBTSO_2_ by direct oxidation of high-purity DBT rather than from refinery sulfone streams, the chemical equivalence of the two products opens an attractive—yet still largely unexplored—opportunity to upcycle ODS-derived sulfone fractions into precursors for optoelectronic materials.

Finally, the polar extraction solvent (acetonitrile, DMF, methanol, ionic liquid) loaded with sulfones is typically regenerated by distillation, back-extraction with water/hexane, or thermal desorption from solid adsorbents (activated carbon, silica, alumina), enabling closed-loop solvent recycling, while the concentrated sulfone fraction is directed to one of the routes above (Thoda et al., 2023).

Despite these advances, the integrated downstream management of ODS-derived sulfones remains a key bottleneck preventing wider industrial deployment of ODS: published process economics studies still rarely include the cost of sulfone disposal or valorization, and a holistic life-cycle assessment of ODS—covering oxidant production, catalyst lifetime, solvent regeneration, and sulfone fate—is still largely missing from the literature. Bridging this gap is essential for fair comparison of ODS with the established HDS technology and for assessing its true sustainability.

The most frequently investigated substrates include dibenzothiophene (DBT), benzothiophene (BT), and alkylated derivatives such as 4,6-dimethyldibenzothiophene (4,6-DMDBT), which are particularly resistant to hydrogenolysis and therefore require more efficient oxidative systems. Alkyl-substituted derivatives of dibenzothiophene, such as 4-methyldibenzothiophene (4-MDBT) and especially 4,6-DMDBT, are characterized by the presence of alkyl groups in positions adjacent to the sulfur atom. These substituents create pronounced steric hindrance that blocks the access of oxidants or catalytic active sites to the sulfur center. As a result, these compounds exhibit the lowest reactivity under ODS conditions (Prins et al., 2006) and are regarded as the most problematic fuel contaminants, being resistant to both hydrodesulfurization (HDS) and mild oxidative treatment.

### 2.2 Factors influencing reactivity and efficiency

The reactivity of sulfur compounds towards oxidation is closely related to the electron density around the sulfur atom. Compounds with higher electron density typically undergo oxidation more rapidly. For instance, the order of reactivity for common sulfur compounds is often 4,6-DMDBT > DBT > BT > Thiophene, correlating with the electron densities on the sulfur atom (Bhutto et al., 2016; Wu et al., 2020). However, steric hindrance introduced by alkyl groups (e.g., methyl groups in 4,6-DMDBT) can sometimes counteract the effect of high electron density, restricting the approach of catalytic active species and thus reducing reactivity.

The extent to which steric effects override electronic effects is strongly system-dependent and is primarily governed by the degree of spatial confinement imposed by the catalyst architecture. In homogeneous or sterically open systems, where the oxidant can freely access the sulfur center, the electronic factor dominates. For instance, Otsuki et al. (Otsuki et al., 2000) demonstrated that in the H_2_O_2_/HCOOH system—where performic acid is generated *in situ* as a compact, sterically undemanding oxidant—the reactivity followed the electron density order: 4,6-DMDBT > 4-MDBT > DBT > BT > thiophenes. A similar trend (4,6-DMDBT > DBT > BT > Th) was observed over MoO_3_/γ-Al_2_O_3_ catalysts with open mesoporous architecture, where pore-diffusion limitations for bulky substrates are minimal (Yin et al., 2011).

In contrast, when the active sites are located within confined pore systems, the methyl substituents at the 4- and 6-positions create a critical steric barrier that reverses the reactivity sequence. For example, functionalized UiO-66(Zr) MOFs (pore aperture ∼6 Å) exhibited the order DBT ≫ 4,6-DMDBT, with 4,6-DMDBT conversion reaching only ∼42% versus ∼82% for DBT under identical conditions (50 °C, 240 min), because the larger 4,6-DMDBT molecule interacts predominantly with the external MOF surface rather than diffusing into the microporous framework (Barghi et al., 2024). Phosphotungstic acid encapsulated within the Zn-based MOF TMU-17-NH_2_ also showed DBT (98%) > 4,6-DMDBT (87%), with the lower conversion of 4,6-DMDBT attributed to its restricted access to the POM active sites inside the three-dimensional framework (Rafiee and Nobakht, 2016). Analogous steric-dominated reactivity sequences (DBT > 4-MDBT >4,6-DMDBT) have been reported for supported Mo/Al_2_O_3_ with t-BuOOH and V_2_O_5_/TiO_2_ with H_2_O_2_ (Yu and Wang, 2013).

These observations highlight that hierarchically porous catalyst architectures—combining micropores with mesopores or macropores—can mitigate steric limitations by providing wider transport channels for bulky substrates while retaining high active-site densities, as demonstrated for PMA-loaded hierarchically porous UiO-66 (PMA/H-UiO-66), which achieved efficient removal of both DBT and 4,6-DMDBT within 20 min (Zong et al., 2023).

An optimal hydrogen peroxide (H_2_O_2_) concentration is crucial for achieving maximum desulfurization efficiency. Excessive H_2_O_2_ can act as a scavenger for hydroxyl radicals, leading to decreased efficiency (Wang and Zhang, 2018). Additionally, high reaction temperatures can cause the self-decomposition of H_2_O_2_, further reducing its effectiveness.

The extent of this non-productive H_2_O_2_ decomposition is, however, strongly governed by the nature of the active metal centre, and the four most commonly employed transition metals in ODS—Fe, Mo, W, and Ti—display markedly different kinetic preferences. Iron-based systems (Fe^2+^/Fe^3+^), due to the facile single-electron redox cycling and the relative weakness of the Fe–O bond, favour homolytic O–O cleavage of H_2_O_2_, generating hydroxyl (•OH) and hydroperoxyl (HO_2_•) radicals via Fenton-type pathways. Although this enables high catalytic activity, it also makes Fe-based systems particularly susceptible to the parallel disproportionation reaction (2 H_2_O_2_ → 2 H_2_O+ O_2_), which becomes increasingly dominant at elevated temperatures or at high H_2_O_2_/Fe ratios. This radical-driven pathway is therefore intrinsically less selective and inherently associated with substantial non-productive oxidant consumption, especially when the substrate is sterically hindered or present at low concentration.

In contrast, the d^0^ early-transition-metal centres Mo(VI), W(VI), and Ti(IV) preferentially activate H_2_O_2_ through heterolytic O–O cleavage, forming stable peroxo (M–η^2^-O_2_) and hydroperoxo (M–OOH) intermediates. These species transfer oxygen atoms electrophilically to the sulfur centre via a two-electron mechanism, bypassing free-radical intermediates and thereby suppressing the parallel disproportionation channel. As a result, Mo- and W-based catalysts—particularly Keggin-type polyoxometalates such as H_3_PW_12_O_40_ and H_3_PMo_12_O_40_, and the Venturello–Ishii anion {PO_4_ [WO(O_2_)_2_]_4_}^3-^ — typically exhibit higher H_2_O_2_ utilization efficiencies (often >70–80% of the H_2_O_2_ consumed is directed toward productive sulfur oxidation) compared with their Fe-based counterparts (often <50%). Titanium (IV) systems (TiO_2_, Ti-MOFs, framework Ti^4+^ sites in TS-1 and Ti-zeolites) likewise operate predominantly through heterolytic activation, producing well-defined Ti–OOH species that are highly selective toward sulfide oxidation; their characteristic Lewis acidity at the Ti centre additionally enhances electrophilic oxygen-atom transfer to the electron-rich sulfur atom.

This fundamental kinetic distinction—radical Fenton-type activation in Fe-based systems versus electrophilic two-electron peroxo-mediated activation in Mo-, W-, and Ti-based systems—accounts for the long-standing dominance of d^0^ early-transition-metal catalysts in industrially relevant ODS processes, where high H_2_O_2_ utilization, selectivity toward sulfones, and minimal non-productive disproportionation are critical performance criteria.

Catalytic performance is heavily influenced by the catalyst’s properties, such as its specific surface area, pore size, active site dispersion, and intrinsic stability. Materials designed with hierarchical porosity or defect engineering can expose more active sites, enhancing catalytic activity. Several operating conditions, including reaction temperature, oxidant-to-sulfur (O/S) molar ratio, catalyst dosage, reaction time, and the choice of solvent or extractant, play crucial roles in optimizing ODS efficiency (Alviar et al., 2024; Khan et al., 2024; Li et al., 2025; Sobati et al., 2010). Careful optimization of these parameters is essential to maximize sulfur removal while minimizing side reactions and reagent consumption. Key advantages of ODS include its mild reaction conditions, as it can be performed at near room temperature and atmospheric pressure, unlike hydrodesulfurization (HDS), which requires high temperatures (290 °C–455 °C) and pressures (10–200 bar) (Hossain et al., 2019). This results in lower operational costs and simpler equipment designs. Additionally, ODS does not consume hydrogen, avoiding the associated costs of hydrogen production and consumption that are significant drawbacks of HDS. ODS is particularly effective for removing refractory sulfur compounds, such as 4,6-DMDBT, which are resistant to conventional HDS due to their molecular structure. Therefore, ODS serves as a potent complementary technique for achieving ultra-deep desulfurization.

Despite the mild operating conditions of ODS, the long-term performance of catalysts is constrained by several universal modes of deactivation that are common across most heterogeneous systems and must be considered alongside reactivity and selectivity.

*Leaching of active metal species* is particularly pronounced when polar extraction solvents (acetonitrile, DMF, methanol) or ionic liquids are used to remove sulfones from the fuel phase: these solvents can solvate and extract weakly anchored metal species (e.g., Mo, W, V, Fe in oxo- or peroxo-forms, as well as polyoxometalate clusters) from the support, leading to progressive loss of active sites and contamination of the polar phase. Leaching is typically diagnosed by hot-filtration tests and ICP-OES/MS analysis of the post-reaction polar phase, and it has been repeatedly reported as the dominant cause of activity loss for impregnated metal-oxide and POM-supported catalysts during repeated cycles (Sun et al., 2019; Zheng et al., 2019).

*Structural collapse of MOF-based catalysts under oxidative conditions* represents another critical limitation. Although Zr-MOFs such as UiO-66 are often promoted as oxidatively stable, prolonged exposure to H_2_O_2_ in protic or aqueous biphasic media can hydrolyze the carboxylate–Zr coordination bonds, oxidize the organic linkers, and induce partial amorphization, particularly in defective frameworks where missing-linker sites are more accessible to oxidative attack (Piscopo et al., 2020; Zhao et al., 2017a; Zhu et al., 2011). The resulting loss of crystallinity, surface area, and pore accessibility translates directly into reduced catalytic activity over successive runs, and is exacerbated by elevated temperature and high O/S ratios.

*Active-site poisoning by strongly adsorbed sulfones* is intrinsic to the ODS chemistry itself: the produced sulfoxides and sulfones (DBTO, DBTO_2_) are markedly more polar than the parent thiophenic substrates and can adsorb tightly on Lewis-acidic active sites (Ti^4+^, Mo^6+^, W^6+^, Zr^4+^) via the S=O group, competitively blocking H_2_O_2_ activation and substrate access (Mortezaee et al., 2021; Yu et al., 2024). This effect is especially pronounced in single-phase (TBHP-based) systems, where sulfones remain in contact with the catalyst; in biphasic H_2_O_2_/acetonitrile systems, continuous extraction of sulfones into the polar phase mitigates but does not eliminate the poisoning.

Additional contributions to deactivation include pore blockage by accumulated polar by-products (water, sulfonic acid residues), coking of organic ligands, and oxidant-induced changes in metal oxidation state. Rational catalyst design therefore requires not only optimization of intrinsic activity but also engineering against these deactivation pathways—through strong covalent anchoring of active metals, post-synthetic modification of MOFs with hydrophobic or defect-healing groups, hierarchical porosity to facilitate sulfone diffusion away from active sites, and regeneration protocols (mild solvent washing, controlled thermal treatment) to remove adsorbed polar species.

### 2.3 Common oxidizing agents

**FIGURE 3:**
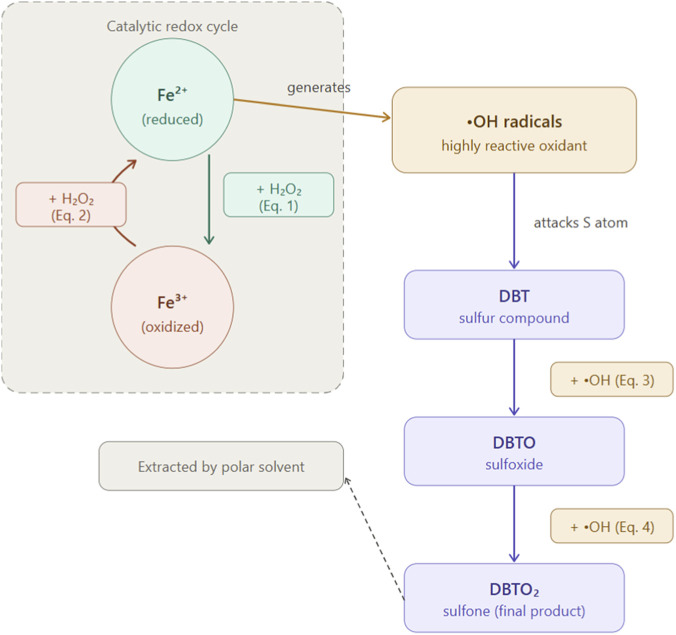
Fenton-like mechanism in H_2_O_2_-based ODS.

Both inorganic (Zhang et al., 2021) and organic oxidants (Liu et al., 2024) have been applied in ODS processes. The choice of oxidant has a significant impact on the reaction rate, selectivity, and catalyst stability. Among them, hydrogen peroxide (H_2_O_2_) is one of the most widely used and preferred oxidants, primarily due to its environmental compatibility, since it generates mainly water and oxygen as by-products (Figure 3). Other commonly employed oxidants include organic hydroperoxides (such as tert-butyl hydroperoxide (TBHP), cumene hydroperoxide (CHP), and cyclohexanone peroxide (CYHPO)), as well as molecular oxygen (O_2_), sodium hypochlorite (NaClO), and ozone (O_3_).

**FIGURE 4:**
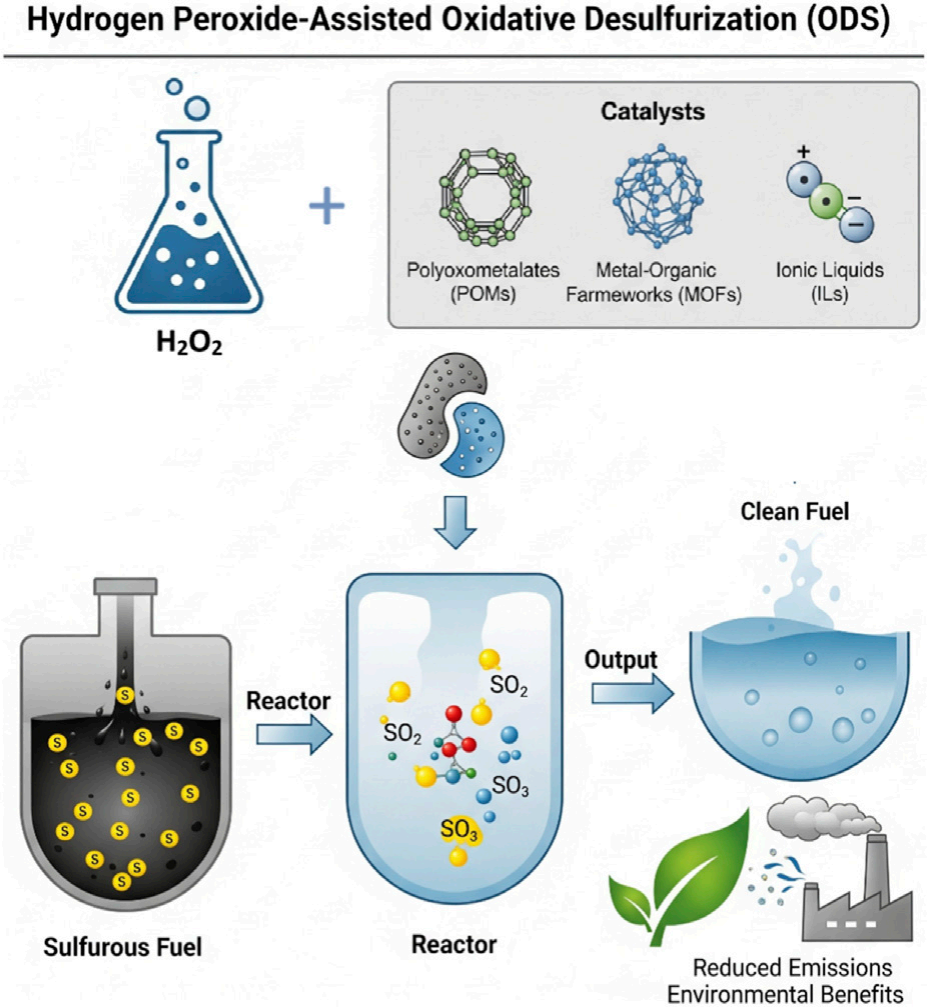
Schematic illustration of hydrogen peroxide-assisted oxidative desulfurization (ODS).

— Percarbonates and peroxo compounds—including sodium percarbonate and meta-chloroperbenzoic acid (m-CPBA), which provide mild reaction conditions and high selectivity (Cha et al., 2010; Jiang et al., 2009; Kubota and Takeuchi, 2004).
— Molecular oxygen (O_2_) — thermodynamically efficient, but requires active catalysts, such as transition metals or coordination complexes, to activate the oxygen molecule (Lü et al., 2007; Lü et al., 2010; Sun et al., 2019; Zhu et al., 2022).
— Organic peroxides (e.g., tert-butyl hydroperoxide, TBHP) — often employed in combination with transition metal catalysts; they exhibit good solubility in the organic phase, which is particularly important for fuel systems (Alcaraz et al., 2023; H et al., 2010; Zaburdaeva and Dodonov, 2011; Lu et al., 2016).
— Hydrogen peroxide (H_2_O_2_) — the most commonly used oxidant owing to its high efficiency, availability, and environmental safety (Figure 4) (Ahmadian and Anbia, 2022; Ibrahim et al., 2017; Jiang et al., 2018; Tong et al., 2024). However, it can decompose at elevated temperatures or in the presence of metals, which reduces its effectiveness.

Hydrogen peroxide (H_2_O_2_) deserves particular attention and is considered in this review as a key oxidant in ODS. This is due to several reasons: it is widely available, environmentally benign, does not generate harmful by-products, and its active species (e.g., •OH or HO_2_•) are capable of efficiently oxidizing a broad spectrum of sulfur-containing compounds. Moreover, H_2_O_2_ is compatible with both homogeneous and heterogeneous catalysts. At the same time, the use of H_2_O_2_ in ODS requires careful consideration of possible interactions with other components of the petroleum matrix. In the presence of unsaturated hydrocarbons, aromatic compounds, and nitrogen-containing fractions, competing oxidations may occur, reducing the selectivity toward the target sulfur-containing molecules. These aspects make the choice of reaction conditions and catalyst critically important for the successful implementation of ODS in real fuel systems. The selection of a specific oxidant ultimately depends on several factors, including the desired selectivity, process safety, cost, and the feasibility of removing by-products (Ahmed et al., 2024; Tahir et al., 2021).

### 2.4 Catalyst selectivity and efficiency: Model fuels versus real commercial fuels

The vast majority of H_2_O_2_-based ODS studies have been conducted using simplified model fuels, typically consisting of individual sulfur compounds (DBT, BT, or 4,6-DMDBT) dissolved in single-component hydrocarbon solvents such as n-octane, n-heptane, or n-dodecane. While such model systems are invaluable for establishing structure–activity relationships and elucidating reaction mechanisms, they do not reflect the complexity of real petroleum matrices, which contain a broad spectrum of competing species including polyaromatic hydrocarbons, olefins, nitrogen-containing heterocycles, and oxygen-containing additives. This compositional complexity can profoundly affect both the selectivity and efficiency of ODS catalysts, and the discrepancy between model and real fuel performance remains one of the key barriers to industrial implementation of ODS technology.

A consistent observation across multiple catalytic systems is that sulfur removal efficiency decreases substantially when transitioning from model to real fuels. For example, the MnO_2_/MrGO composite catalyst reported by Ahmad et al. (Ahmad et al., 2021) achieved approximately 80% DBT conversion in model fuel, whereas the same system under optimized conditions yielded only 67.8%, 59.5%, and 51.9% sulfur removal from real diesel, gasoline, and kerosene, respectively. The PTA@MIL-101(Cr)-NH_2_ system reported by Wang et al. (Wang et al., 2014) achieved 100% DBT removal in model fuel but showed reduced performance when tested with real gasoline, as noted by Wang et al. (Wang et al., 2017) who observed that the window size of the MOF host plays a critical role in accommodating the diverse molecular sizes present in commercial fuels. Ahmed et al. (Ahmed et al., 2022) reported that the dicarboxylic acid/H_2_O_2_ system could reduce sulfur content in real high-sulfur diesel, although the efficiency was lower than in model systems due to the presence of competing substrates. Mortezaee et al. (Mortezaee et al., 2021) confirmed that the H_2_O_2_/formic acid system applied to a real mineral lubricant base oil also exhibited reduced efficiency compared to model DBT solutions.

Several factors account for this performance gap. First, nitrogen-containing heterocycles such as indole, quinoline, and carbazole are known to competitively consume H_2_O_2_ and reactive oxygen species, thereby reducing the amount of oxidant available for sulfur compound oxidation. Otsuki et al. (Otsuki et al., 2000) demonstrated that the addition of indole significantly retarded DBT oxidation in the HCOOH/H_2_O_2_ system. This finding has been corroborated by studies on silica gel-based ODS systems, where nitrogen compounds were identified as the primary inhibitors of desulfurization, even at concentrations as low as a few hundred ppm (Wu et al., 2019). Second, polyaromatic hydrocarbons (e.g., phenanthrene, naphthalene) can compete with sulfur compounds for adsorption on catalyst active sites or pore space, particularly in microporous materials such as MOFs and zeolite-supported catalysts (Xiao et al., 2012). Third, olefinic compounds present in fuels derived from fluid catalytic cracking (FCC) can undergo epoxidation or other side reactions with H_2_O_2_, leading to non-productive oxidant consumption and reduced selectivity toward sulfur species (Otsuki et al., 2000). Fourth, water generated as a by-product of H_2_O_2_ decomposition or formed during the reaction can poison Lewis acid sites on the catalyst surface, diminishing catalytic activity over time.

It is noteworthy that certain catalyst systems show a less pronounced performance gap. The extractive-catalytic ODS (ECODS) approach, where the oxidation catalyst is combined with ionic liquid or DES-based extraction phases, can partially mitigate the matrix effect. For example, the [Hnmp]ClxFeCl_3_ ionic liquid system described in (Hao et al., 2019) demonstrated dual extraction-catalytic functionality, which inherently pre-concentrates sulfur compounds in the IL phase before oxidation, thus reducing interference from bulk hydrocarbons. Additionally, the POM-based systems (Choi et al., 2016), which operate through oxygen transfer via peroxo-metal intermediates rather than free radical pathways, may exhibit greater selectivity toward sulfur compounds in the presence of aromatic and olefinic competitors, since the electrophilic oxygen transfer is preferentially directed toward the high-electron-density sulfur atoms.

In summary, the transition from model to real fuel applications necessitates careful consideration of matrix effects, particularly the presence of nitrogen heterocycles and polyaromatics. Future catalyst design should focus on enhancing substrate selectivity through molecular recognition strategies, pore-size engineering, and the development of catalytic systems that minimize non-productive H_2_O_2_ decomposition in complex fuel environments.

### 2.5 Engineering challenges for industrial-scale implementation of H_2_O_2_-assisted ODS

While the majority of H_2_O_2_-based ODS studies have established proof-of-concept performance at the laboratory scale, the translation of these systems to industrially relevant capacities raises a number of chemical engineering challenges that must be addressed before commercial deployment. Three issues are particularly critical: liquid–liquid mass transfer limitations in biphasic oil–aqueous systems, the safe handling of large volumes of concentrated H_2_O_2_, and the transition from batch to continuous-flow operation.

H_2_O_2_-assisted ODS typically operates as a biphasic system in which the sulfur substrate resides in the non-polar fuel phase while H_2_O_2_ and many catalytic species reside in the polar (aqueous, IL, or DES) phase. The overall rate of sulfur oxidation is therefore frequently controlled not by the intrinsic catalytic kinetics, but by the interfacial transport of the substrate, the oxidant, and the oxidized products across the phase boundary. Several engineering strategies have been proposed to mitigate this limitation, including high-shear mixing-assisted oxidative desulfurization (MAOD), in which intense agitation (10,000–18,000 rpm) generates fine emulsions with very high interfacial area (Cha et al., 2010; Peng et al., 2018; Wang et al., 2014; Wang et al., 2017; Zhang et al., 2016); ultrasound-assisted ODS, where acoustic cavitation continuously renews the interface and locally generates additional reactive species (de Luna et al., 2018a; Liu et al., 2018; Rafiee and Nobakht, 2016); microwave-assisted ODS in helical-coil and other intensified reactors (De Luna et al., 2018b; Ivanin et al., 2020); and the use of amphiphilic or surfactant-type catalysts (e.g., long-chain alkyl-imidazolium phosphotungstates [C_16_MIM]_3_PW_12_O_40_, decatungstate-based emulsifiers), which self-assemble at the oil–water interface and act as “mobile” reaction sites that bypass the mass-transfer barrier altogether (Khan et al., 2024; Maurya et al., 2010; Zhang et al., 2021). For industrial-scale operation, however, none of these intensification approaches is straightforwardly scalable: ultrasonic and microwave reactors face well-known limitations in penetration depth and power density when scaled to refinery throughputs, while high-shear mixers entail substantial energy consumption and emulsion-breaking costs downstream. Rational reactor design—including microstructured contactors, static mixers, oscillatory baffled reactors, and membrane contactors—is therefore an active area of research that deserves systematic attention in the ODS context (Granadeiro et al., 2016).

Although H_2_O_2_ is widely regarded as a “green” oxidant because its only by-products are water and oxygen, its industrial-scale handling is far from trivial. Commercial H_2_O_2_ is supplied at concentrations of 30–70 wt%, and concentrations above ∼35 wt% are classified as hazardous oxidizers under DOT, UN, and CLP regulations. Several specific hazards must be engineered against: exothermic self-decomposition catalyzed by trace transition-metal ions (Fe, Mn, Cu, Co) — the very same ions that often serve as ODS catalysts—which can lead to runaway pressure build-up in closed vessels; explosion hazards associated with the contact of concentrated H_2_O_2_ with organic matter, particularly hydrocarbons of the fuel matrix itself, where local hot spots may ignite peroxide–hydrocarbon mixtures; and corrosion of standard refinery metallurgy, requiring passivated stainless steel (electropolished 316L, 904L) or aluminum alloys, dedicated transfer lines, and breather-vented storage. Best practice in ODS scale-up therefore advocates for the use of organic-peroxide precursors (TBHP, performic acid generated *in situ* from HCOOH + H_2_O_2_) that present a lower hazard profile while preserving comparable oxidation activity, or for the on-site generation of dilute H_2_O_2_ at the point of use, which eliminates the safety risks associated with bulk H_2_O_2_ transport and storage.

The vast majority of laboratory ODS studies have been performed in stirred batch reactors with reaction times ranging from 30 min to several hours. Industrial refineries, however, operate continuously, and a viable ODS unit must be integrated into a continuous process stream with steady-state residence times measured in minutes. Continuous-flow ODS reactors offer several intrinsic advantages over batch systems: more precise control of residence time and temperature profiles, improved heat removal that suppresses non-productive H_2_O_2_ decomposition, smaller hold-up of hazardous reagents (consistent with the inherent-safety principle), and easier coupling with downstream separation (continuous LLE columns, adsorption beds, sulfone decomposition reactors). Several reactor configurations have been explored experimentally for continuous H_2_O_2_-based ODS, including sonochemical flow and loop reactors capable of treating diesel feeds with substantial sulfur and nitrogen reduction (de Luna et al., 2018a; Liu et al., 2018); continuously mixed reactor configurations operating in oxidative-adsorptive mode for diesel fuel (Granadeiro et al., 2016); microwave-assisted helical coil reactors with experimentally validated CFD models (De Luna et al., 2018b); and hybrid extractive–catalytic (ECODS) flow systems where IL or DES phases circulate in a closed loop, simultaneously extracting sulfur substrates and catalyzing their oxidation. Despite these promising developments, only a handful of continuous-flow ODS demonstrations have been reported at pilot scale, and a robust long-term operational dataset (catalyst lifetime over hundreds of hours, sulfur breakthrough behavior, integrated solvent and sulfone management) is still largely missing from the open literature. Closing this gap is essential before H_2_O_2_-assisted ODS can credibly compete with—or complement—entrenched HDS technology at refinery scale.

In summary, the chemical engineering aspects of ODS scale-up—interfacial mass transfer, oxidant safety, and continuous-flow reactor design—are at least as important as the catalytic chemistry itself in determining the industrial viability of the technology. Future research should explicitly integrate reactor and process engineering considerations into catalyst development, rather than treating them as downstream concerns.

## 3 Challenges in oxidation mechanisms

Understanding the mechanism of H_2_O_2_-assisted desulfurization (ODS) is crucial for process optimization, yet it poses a number of serious challenges due to its complexity. The key mechanistic issues can be summarized as follows: H_2_O_2_ can decompose or be activated to generate various reactive oxygen species (ROS), including hydroxyl radicals (•OH), hydroperoxyl radicals (•OOH), peroxo species (O_2_^2-^), superoxide (O_2_•^-^), or even singlet oxygen (^1^O_2_). The specific dominant ROS largely depends on.

### 3.1 Catalyst type

Catalysts play a crucial role in the efficiency of oxidative desulfurization (ODS) systems, working synergistically with the oxidant to facilitate the oxidation of organic sulfur compounds. They enhance the generation of reactive oxygen species (ROS) and accelerate the oxidation process. Various types of catalysts are employed in ODS, each contributing to improved catalytic performance:

Polyoxometalates (POMs) (Bryzhin et al., 2019; Craven et al., 2018) are highly effective due to their tunable properties, exposed active sites, and excellent catalytic performance in redox reactions. POMs are often combined with carriers to create composite materials, such as encapsulated POMs, which enhance both stability and catalytic activity.

Metal-Organic Frameworks (MOFs) (Luo et al., 2021) are used in ODS due to their structural designability, tunable properties, and high specific surface area. MOFs significantly enhance catalytic desulfurization, with the pore size and active metal centers (such as V, Fe, Zr, and Ce) influencing catalytic performance and the diffusion of reactants.

Ionic Liquids (ILs), a class of molten salts, are extensively used in ODS for their stable state, structural flexibility, and ability to modulate properties (Hussain et al., 2023; Tarkhanova et al., 2023a). Acidic ILs can function both as extractants and catalysts, decomposing H_2_O_2_ to generate hydroxyl radicals.

Deep Eutectic Solvents (DESs) are emerging as environmentally friendly alternatives to ILs due to their ease of synthesis, low cost, and environmental benefits. DESs can act as both catalysts and reaction media, often by oxidizing organic acids within the solvent to form peroxy acids, which then oxidize sulfur compounds.

Metal-based Catalysts include various metal oxides, such as MoO_3_, WO_3_, CuO, MgO, Fe_2_O_3_, and MnO_2_, as well as bimetallic composites (Bakar et al., 2012; Bryzhin et al., 2020a; Muhammad et al., 2018; Teimouri et al., 2018). High-site-density catalysts are often supported on metal oxide surfaces to enhance dispersion, prevent agglomeration, and improve activity and selectivity. Examples include AgO/ZnO/HY-Zeolite, Fe-promoted Co-Mo/Al_2_O_3_, and Ni-Mo/Al_2_O_3_.

Carbon-based Materials are commonly used in catalysis, with both physical and chemical methods employed to optimize their performance. Materials such as graphene oxide, carbon nanotubes, and nitrogen-doped carbon are significant for fuel oxidation and desulfurization. Notably, hexagonal boron nitride (h-BN) has been explored as a metal-free catalyst, demonstrating high efficiency in desulfurizing refractory sulfur compounds.

Each of these catalysts plays a pivotal role in optimizing the ODS process, contributing to higher sulfur removal efficiencies and enabling the production of ultra-low sulfur fuels.

To provide a more quantitative basis for comparing the catalytic systems discussed above, Table 1 summarizes the principal performance metrics reported for representative POM-, MOF-, IL-, and homogeneous metal-complex-based catalysts in ODS processes. The compiled parameters include the optimal reaction temperature, oxidant-to-sulfur (O/S) molar ratio, initial sulfur content, catalyst loading, reaction time, and sulfur conversion. It should be noted that intrinsic kinetic descriptors such as Turnover Frequency (TOF) and activation energy (E_a_) are inconsistently reported across the ODS literature, which limits a fully uniform kinetic benchmarking; therefore, the table reflects the quantitative data explicitly available in the corresponding references.

**TABLE 1:** Oxidative desulfurization activity of different catalysts (first part) under different optimized reaction conditions.

Catalysts	Temp. (K)	Conv. (%)	O/S ratio	Initial S Content	Catalyst amount	Reaction time	Ref.
[CNT/PVPNO]/MTO	333	>99	4	9200 ppm	0.276 g/L	180 min	Piccinino et al. (2017)
PS-[VVO2(fsal-dmen)]	333	98.4	3	1500 ppm	7.5 g/L	120 min	Maurya et al. (2010)
[C4mim]3Fe(CN)6^a^	313	97.9	4	250 ppm	6 g/L	300 min	Jiang et al. (2014)
[WO(O2)2Phen]H2O	343	98.6	10	1000 ppm	0.411 g/L	180 min	Zhu et al. (2007)
VO(acac)_2_^a^	303	99.6	5	500 ppm	0.216 g/L	120 min	Zhu et al. (2013)
TiO2/porous glass	333	100	10	150 ppm	9.85 g/L	2 min	Shen et al. (2016)
MoO(O2)2.gly^a^	343	99.2	4	1000 ppm	8 g/L	180 min	Zhu et al. (2011)
FeIIITPP(PF6)^a^	333	99.5	3	500 ppm	22 g/L	240 min	Zhao et al. (2017a)
(PorMe4)FeCl	333	100	4	500 ppm	20 g/L	300 min	Zhao et al. (2017b)
µ-O(FeTPFPP)_2_	Room Temp	100	12	500 ppm	1.5 g/L	180 min	Aguiar et al. (2014)
Zr-MOF-808	313	100	5	1000 ppm	2 g/L	5min	Zheng et al. (2019)
Zr-UiO-66D	323	100	95	500 ppm	3 g/L	30 min	Granadeiro et al. (2015)
Ti-UiO-66	333	91.7	6	1000 ppm	4 g/L	120 min	Ye et al. (2017)
PTA@MOF-808A	333	100	5	1000 ppm	7.5 g/L	30 min	Lin et al. (2018)
2PTA@MIL-53(Al)-NH2	328	100	20	500 ppm	4 g/L	300 min	Zhang et al. (2016)
PTA@UiO-67	343	100	13	1000 ppm	2 g/L	60 min	Peng et al. (2018)
POM@MIL-53(Al)-NH2	343	99.9	23	2348 ppm	3 g/L	120 min	Granadeiro et al. (2016)
PTA@MIL-101(Cr)-NH2	323	100	4	100 ppm	4 g/L	60 min	Wang et al. (2014)
16%PTA@MIL-100(Fe)	343	100	4	950 ppm	∼7.5 g/L	60 min	Wang et al. (2017)
Activated carbon/PTA	336	62.73	∼1	2.3 wt%	∼1 g/L	88.5 min	Barilla et al. (2022)
Calcinated PTA	323	100	1	500 ppm	∼1 g/L	30 min	Wan et al. (2019)
PTA	343	85.90	0.6	1480 ppm	8 g/L	∼3 min	de Luna et al. (2018a)
PTA	313	100	1	1428 ppm	8 g/L	​	De Luna et al. (2018b)
Sodium phosphotungstate	343	94.8	1	500 ppm	N/A	30 min	Choi et al. (2016)
H_3_PW_12_O_40_ (PTA) H_3_PMo_12_O_40_ (PMo) H_4_SiW_12_O_40_ (SiW)	343	>90 >70 <40	1	500 ppm	N/A	30 min	Yang et al. (2023)

As can be seen from Table 1, the majority of the surveyed catalytic systems achieve near-complete sulfur conversion (>95%) under relatively mild conditions (303–343 K), confirming the general feasibility of ODS as a low-temperature alternative to HDS. Among the homogeneous metal-complex catalysts, vanadium- and molybdenum-based systems such as VO(acac)_2_ and MoO(O_2_)_2_·gly demonstrate high activity at moderate O/S ratios (4–5), indicating efficient peroxo-mediated oxygen transfer. MOF-based and POM@MOF composite catalysts (Zr-MOF-808, PTA@MOF-808, PTA@UiO-67, PTA@MIL-101(Cr)-NH_2_) generally reach full conversion within 5–60 min, reflecting the synergistic effect between the porous MOF support and the embedded polyoxometalate active phase. In contrast, unsupported heteropolyacids show pronounced differences in activity (PTA > PMo > SiW) under identical conditions, highlighting the role of the heteroatom and addenda metal in determining catalytic performance. The required O/S ratio also varies considerably across the surveyed systems—from ∼1 for heteropolyacid-based catalysts to >20 for some MOF-supported POMs—which directly affects the practical and economic feasibility of each approach. Overall, the comparison indicates that supported POM systems and peroxo-forming transition-metal complexes currently offer the most favourable balance between activity, selectivity, and operating conditions among the catalyst classes considered.

### 3.2 Additional parameters Affecting the efficiency of H_2_O_2_-Based oxidative desulfurization

The efficiency of H_2_O_2_-based oxidative desulfurization (ODS) is influenced by several critical factors, including pH, solvent properties, and advanced techniques aimed at enhancing the process. In acidic conditions, electrophilic oxygen species are favored, while basic conditions promote nucleophilic oxygen species. Neutral pH can lead to the generation of radical species, which may facilitate radical-based oxidation mechanisms (Yang et al., 2023). Solvent properties, such as polarity and whether the solvent is protic or aprotic, also play a key role in the stability and reactivity of reactive oxygen species (ROS) (Mitroka et al., 2010). The choice of solvent impacts the overall effectiveness of the reaction by stabilizing intermediates and enhancing the reactivity of ROS. Beyond the development of catalysts, several methods can further improve the efficiency of ODS. Ultrasonication, or cavitation, enhances mass transfer and generates reactive species such as hydroxyl and hydrogen radicals through pyrolytic effects and the decomposition of the oxidant (Jalali and Sobati, 2017). This improves micro-mixing and emulsification, increasing the contact area between the phases and accelerating the desulfurization process. Microwave irradiation has also been shown to boost sulfur removal by selectively heating polar components in the reaction mixture, thus speeding up the reaction kinetics (Kaeed et al., 2024). These techniques make ODS a versatile and effective method for reducing sulfur content in fuels, supporting cleaner energy production. The fundamental principle behind H_2_O_2_-based ODS lies in the activation of hydrogen peroxide to generate highly reactive oxygen species (ROS), which selectively oxidize organic sulfur compounds found in fuels. Sulfur compounds are typically oxidized in a sequential manner. For example, dibenzothiophene (DBT) is first converted into dibenzothiophene oxide (DBTO), a sulfoxide, which is then further oxidized into dibenzothiophene sulfone (DBTO_2_), a sulfone (Otsuki et al., 2000; Wu et al., 2019; Xiao et al., 2012; Zhang et al., 2011). The step that generates the sulfoxide from dibenzothiophene is often the rate-limiting reaction in the process. This oxidation results in a significant increase in the polarity of the sulfur-containing molecules, making it easier to separate them from the non-polar hydrocarbon fuel phase through extraction or adsorption. This enhanced polarity of the oxidized sulfur species plays a crucial role in the effective removal of sulfur from fuels, further optimizing the desulfurization process.

### 3.3 H_2_O_2_ activation mechanisms

The efficiency of H_2_O_2_-assisted oxidative desulfurization (ODS) is largely determined by the activation of hydrogen peroxide to generate potent oxidizing agents. Several mechanisms are employed to activate H_2_O_2_ and enhance its oxidation capabilities. In Fenton and Fenton-like systems, H_2_O_2_ reacts with transition metal ions or metal oxides, such as Fe^2+^/Fe^3+^, Co^2+^, Cu^2+^, Ce3+/Ce4+, ZrO2, MoO3 or V2O3, to generate highly reactive hydroxyl radicals (•OH) that play a key role in the oxidation of sulfur compounds (Bryzhin et al., 2020b; Ivanin et al., 2020; Tarkhanova et al., 2019; Tarkhanova et al., 2023b). For example, a FeCeOx/H_2_O_2_/TP (tea polyphenols) system has been shown to effectively generate abundant hydroxyl radicals, with the addition of TP significantly promoting the catalytic activity of FeCeOx in H_2_O_2_ decomposition (Liu et al., 2023). Similarly, cobalt ferrite (CoFe_2_O_4_) nanoparticles, known for their catalytic activity, can activate peroxymonosulfate (PMS) to produce sulfate radicals and hydroxyl radicals, enhancing the oxidation of dibenzothiophene (DBT). Additionally, Fe_2_(MoO_4_)_3_ catalysts can activate gas-phase H_2_O_2_ to produce hydroxyl radicals for the oxidation of pollutants (Liu et al., 2018). The synergistic interaction between metal components, such as Fe and Co in CoFe_2_O_4_, further boosts catalytic activity (Table 1) (Xu et al., 2024b).

Another activation pathway involves the formation of peroxyacids in systems containing carboxylic acids, such as formic acid (HCOOH) and acetic acid (CH_3_COOH), in combination with H_2_O_2_. These peroxyacids, such as performic acid, act as powerful oxidants for sulfur compounds (Ahmad et al., 2021). The acidity of the medium can also enhance catalyst activity by promoting H_2_O_2_ adsorption. For instance, sulfuric acid and formic acid can increase catalytic efficiency due to their enhanced acidity (Sachdeva and Pant, 2010).

In addition to radical mechanisms, some catalysts, particularly polyoxometalates (POMs), can activate H_2_O_2_ to form peroxo-POM species. These species directly transfer oxygen atoms to sulfur compounds, facilitating their oxidation through a non-radical mechanism (Komintarachat and Trakarnpruk, 2006). Bismuth molybdate catalysts, for example, facilitate oxidation via oxygen bridging between two metal atoms in the lattice oxide, which serves as the active center for redox reactions. In these systems, desulfurization is less influenced by hydroxyl or superoxide radical scavengers, indicating that a non-radical pathway is at play (Zhang et al., 2023). The high electron density of sulfur atoms in DBT makes them highly susceptible to attack by these oxygen-containing groups, enhancing the efficiency of the desulfurization process (Sun and Wang, 2025).

Ultrasonication, or cavitation, is another technique employed to enhance ODS efficiency. The application of ultrasound induces cavitation, which intensifies mass transfer between immiscible phases and generates reactive species such as hydroxyl and hydrogen radicals (•OH, •H) through pyrolytic effects and the decomposition of oxidants (Baradaran and Sadeghi, 2019). Cavitation can directly dissociate H_2_O_2_ into hydroxyl radicals, thus boosting the oxidation potential and improving the overall efficiency of sulfur removal. Finally, photocatalysis also plays a role in enhancing ODS. UV/H_2_O_2_ systems are known to generate hydroxyl radicals (•OH), and photocalysts such as TiO_2_ and graphene/porphyrin composites can activate H_2_O_2_ by leveraging photogenerated electrons and holes to produce reactive oxygen species (ROS), further promoting the oxidation of sulfur compounds (Zhou et al., 2023). This combination of advanced techniques improves the overall efficiency of H_2_O_2_-assisted ODS, offering a promising approach for achieving ultra-low sulfur content in fuels.

### 3.4 Mechanistic insights into H_2_O_2_-Based Fenton-like desulfurization systems

The Fenton mechanism is a well-known advanced oxidation process involving the reaction of hydrogen peroxide (H_2_O_2_) with iron (Fe^2+^) to generate highly reactive hydroxyl radicals (•OH). These radicals are capable of oxidizing a wide range of organic and inorganic pollutants. In oxidative desulfurization (ODS), the Fenton process is applied to convert organosulfur compounds into more polar sulfoxides and sulfones, which can then be readily removed by extraction or adsorption. This mechanism is characterized by the high oxidative potential of •OH radicals, operation under mild temperature and pressure conditions, simplicity and cost-effectiveness compared to hydrodesulfurization (HDS), and efficiency against refractory sulfur compounds such as dibenzothiophene derivatives.

In the study (Hao et al., 2019), the catalytic oxidative desulfurization (ODS) mechanism of dibenzothiophene (DBT) using Lewis acidic ionic liquids (ILs) based on ferric chloride—specifically [Hnmp]Cl∙xFeCl_3_—in the presence of hydrogen peroxide (H_2_O_2_) was investigated in detail. Gas chromatography–mass spectrometry (GC–MS) analysis of the reaction products revealed two main peaks corresponding to the starting DBT and its sulfone (DBTO_2_, m/z = 216.0), demonstrating the high selectivity of the system toward the target product and the absence of by-products in the organic phase.

A key aspect of the study was the identification of reactive oxygen species (ROS) involved in the oxidation process. Radical-trapping experiments established that the hydroxyl radical (•OH) played the primary role in DBT oxidation, whereas the contribution of the superoxide anion (O_2_•^-^) was minimal. Electron paramagnetic resonance (EPR) confirmed the formation of both radicals in the IL/H_2_O_2_ systems, with the intensity of the •OH signal correlating with the observed catalytic activity (Liu et al., 2021).

**FIGURE 5:**
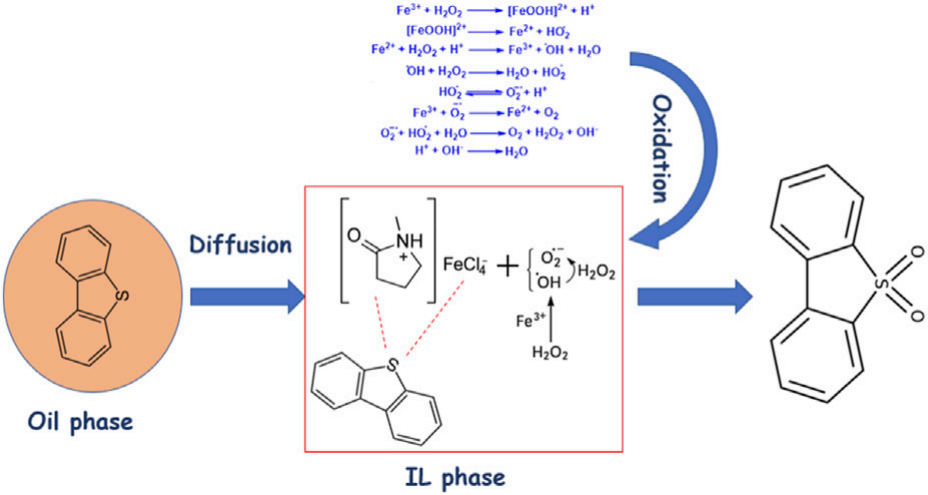
Proposed mechanism scheme.

The radical generation mechanism involves several sequential steps, including the formation of [FeOOH]^2+^ from Fe^3+^ and H_2_O_2_, its reduction to Fe^2+^ with generation of HO_2_•, and subsequent •OH formation via a Fenton-type reaction. The main oxidation of DBT to DBTO_2_ occurs in the ionic liquid phase, where Fe^3+^ coordinates with the substrate, facilitating its activation (Hao et al., 2019). The authors also carried out radical inhibition experiments using benzoquinone and isopropanol and performed EPR analyses of the reaction mixtures. The results indicated a radical pathway involving hydroxyl radicals and superoxide species. This led to the proposal of a DBT oxidation mechanism in the presence of supported ionic derivatives (Figure 5). Initially, the substrate transfers into the IL phase, after which active radicals are generated, with •OH identified as the main oxidant. This conclusion was further supported by IR spectroscopy analysis of the catalyst structure before and after the reaction.

A control experiment using an aqueous FeCl_3_ solution (outside of the IL medium) showed only minor sulfur removal, confirming that the IL environment is essential for the efficient activation of H_2_O_2_ and the generation of reactive oxygen species. Furthermore, the [Hnmp]Cl∙1.0FeCl_3_-based IL exhibited high stability toward •OH radicals and demonstrated good recyclability: after four cycles, the reaction maintained high efficiency (Ali et al., 2023). Thus, the study demonstrates that FeCl_3_-based ILs play a dual role—acting both as an extraction medium and as a catalyst generating reactive radicals, with •OH being the primary oxidant. Their high selectivity, stability, and reusability make these systems promising candidates for environmentally friendly and efficient desulfurization of liquid fuels.

In another study (Ali et al., 2023), a system based on deep eutectic solvents (DES) demonstrated high efficiency in the oxidative desulfurization (ODS) of sulfur-containing compounds such as dibenzothiophene (DBT). The proposed mechanism involves a two-step process, determined by the immiscibility of DES with the model hydrocarbon phase. In the first step, DBT is extracted from the oil phase into the DES phase, and in the second step, it is oxidized to the sulfone.

A DES containing Fe^3+^ ions (e.g., FeCl_4_^−^), in the presence of H_2_O_2_, functions analogously to a Fenton system, generating highly reactive hydroxyl radicals (•OH). These radicals sequentially oxidize DBT first to sulfoxide and then to sulfone. The uniqueness of this approach lies in the fact that DES not only serves as an extraction medium but also actively participates in the catalytic process. Among the systems investigated, the Fe-based DES exhibited the highest catalytic activity when H_2_O_2_ was used as the oxidant. In addition to its high efficiency, this system showed remarkable stability and could be reused multiple times without significant loss of activity, making it economically viable and suitable for practical fuel desulfurization applications.

**FIGURE 6:**
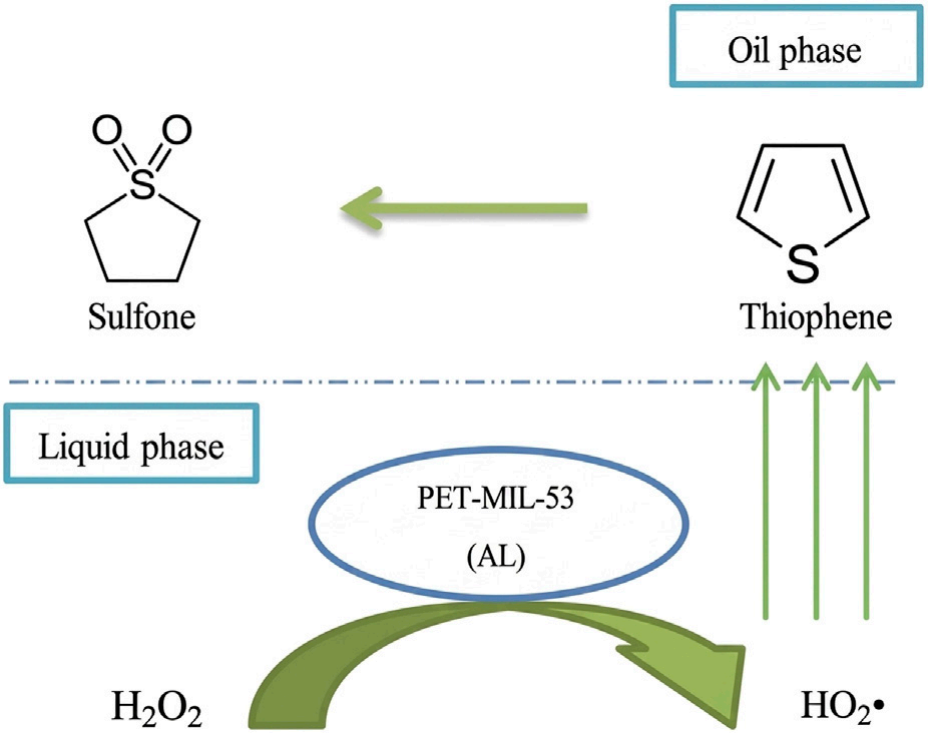
Schematic representation of oxidative desulfurization of thiophene using H_2_O_2_ and PET-MIL-53(Al) catalyst.

Another efficient catalyst for oxidative desulfurization (ODS) is PET MIL-53 (Al)—an aluminum-based metal–organic framework synthesized from recycled plastic waste (PET bottles). According to the proposed mechanism (Figure 6), upon interaction of hydrogen peroxide (H_2_O_2_) with Brønsted acid sites on the PET MIL-53 (Al) surface, active hydroperoxyl radicals (HO_2_•) are generated. These radicals subsequently oxidize thiophene into the corresponding sulfones. Due to their high polarity, the oxidation products (sulfones) can be easily extracted from the model hydrocarbon phase using a polar solvent such as acetonitrile (Pan et al., 2024).

To confirm the participation of •OH radicals in the reaction, ATR-FTIR measurements were performed before and after the addition of H_2_O_2_. The increase in the intensity of O–H stretching bands in the spectrum after the reaction onset confirmed the formation of hydroxyl radicals, which play a key role in the catalytic process (Pan et al., 2024).

In the following study, a detailed mechanistic investigation was conducted on various catalytic oxidative desulfurization (ODS) systems of dibenzothiophene (DBT) employing hydrogen peroxide (H_2_O_2_) and Fe^2+^ ions under ultrasonic irradiation (sonolysis), Fenton, and photo-Fenton processes, as well as within the Fe(II)-oxalate system. Particular attention was devoted to the generation of hydroxyl radicals (•OH), which play a key role in the deep oxidation of DBT to the corresponding sulfone (Chakrabarty et al., 2021).

The experiments were categorized into six systems: (i) pure sonolysis (US), (ii) mechanical stirring + H_2_O_2_, (iii) US + H_2_O_2_, (iv) US + H_2_O_2_ + Fe^2+^ (Fenton), (v) US + H_2_O_2_ + Fe^2+^ + UV (photo-Fenton), and (vi) US + H_2_O_2_ + Fe^2+^ + oxalate (Fe(II)-oxalate system). The results showed that mechanical stirring with H_2_O_2_ led to less than 1% DBT removal, confirming the weak oxidative capacity of H_2_O_2_ without an activator. Meanwhile, pure sonolysis achieved only ∼4.5% desulfurization despite radical generation under cavitation conditions. The addition of H_2_O_2_ during sonolysis increased the reaction rate, but the overall efficiency remained low (∼6%), which was attributed to the low partial pressure of H_2_O_2_ and its limited transfer into cavitation bubbles (Lü et al., 2015).

A significant increase in efficiency (∼93%) was observed upon the addition of Fe^2+^ ions to the US + H_2_O_2_ system, which activated the Fenton reaction: Fe^2+^ + H_2_O_2_ → Fe^3+^ + •OH + OH^−^. The hydroxyl radicals generated in this reaction initiate the oxidation of sulfur-containing compounds.

**FIGURE 7:**
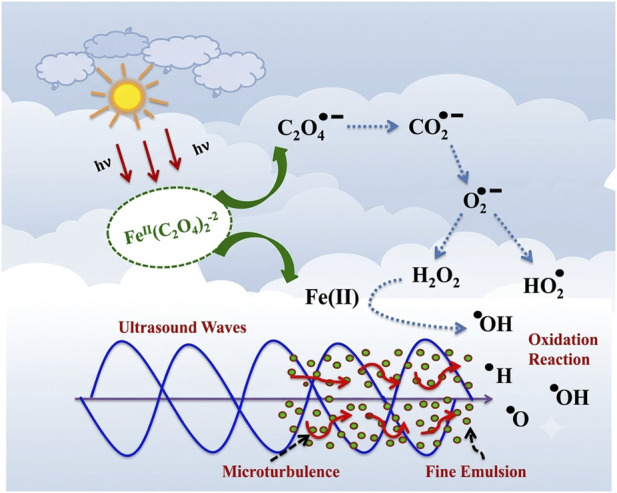
Schematic illustration of a photo-assisted sono-Fenton-like system.

The application of the photo-Fenton approach with UV irradiation, contrary to expectations, did not provide additional improvement in efficiency. This can be explained by the fact that UV light, on the one hand, promotes the photogeneration of •OH through the photoreduction of Fe^3+^ (Fe(OH)^2+^ + hυ → Fe^2+^ + •OH), but on the other hand, it also induces undesirable side processes (Equations 8, 9), including radical recombination and their scavenging by excess H_2_O_2_ (Figure 7).

$$•\text{OH}+H_{2}O_{2}\to H_{2}O+{\text{HO}}_{2}•$$ (8)

$$•\text{OH}+{\text{HO}}_{2}•\to H_{2}O+O_{2}•$$ (9)

Thus, an excess of radicals can lead to their annihilation, thereby reducing the overall efficiency. A comparison between the US + H_2_O_2_ + Fe^2+^ and the US + H_2_O_2_ + Fe^2+^ + oxalate systems revealed a similar degree of desulfurization; however, the oxalate system provided higher reaction kinetics. This was attributed to the formation of reactive Fe(II)-oxalate complexes, such as Fe(C_2_O_4_) and Fe(C_2_O_4_)_2_^2-^, which exhibit faster radical generation compared to free Fe^2+^/Fe^3+^ ions (Lü et al., 2015).

In summary, this study demonstrates that efficient catalytic desulfurization of DBT is feasible via radical pathways activated by the combination of H_2_O_2_ and iron ions, while also confirming that excessive radical generation or the presence of UV irradiation does not necessarily enhance efficiency due to competing side processes.

As discussed above, recent developments and trends are focused on designing modified Fenton systems employing heterogeneous catalysts (e.g., Fe_3_O_4_, iron-based metal–organic frameworks). Enhanced radical generation can also be achieved using light or electricity, in so-called photo- and electro-Fenton processes. Furthermore, combined ODS approaches have been implemented, such as Fenton coupled with ultrasound, microwaves, or ionic liquids (Brillas and Martínez-Huitle, 2015).

Synthesized tetrabutyl titanate (TiO_2_-TBOT), exhibited significantly higher activity in oxidative desulfurization (ODS) compared to TiO_2_-TTIP, despite their similar BET surface areas (165 m^2^/g and 133 m^2^/g, respectively). This observation indicates the presence of an additional mechanism underlying the observed catalytic performance. The proposed mechanism involves the formation of active Ti–peroxo species through the interaction of H_2_O_2_ with coordinatively unsaturated Ti^4+^ sites on the surface of TiO_2_-TBOT.

According to the literature, such Ti–peroxo species are capable of transferring an oxygen atom (*[O]) to the substrate, similar to the epoxidation mechanism of alkenes. In the case of dibenzothiophene (DBT), the active Ti–peroxo species first extract DBT molecules into the extracting phase (e.g., methanol), where DBT undergoes sequential oxidation to sulfoxide (DBTO) and then to sulfone (DBTO_2_). The high polarity of the final product (DBTO_2_) facilitates its extraction and removal from the model fuel.

The formation of Ti–hydroperoxo species was confirmed by FT-IR and Raman spectroscopy, while the presence of acidic sites was demonstrated by NH_3_-TPD analysis. The catalytic cycle is sustained through the reversible Ti^4+^ ⇌ Ti^3+^ transition, which ensures the regeneration and stability of active sites. Structural modeling further demonstrated that the presence of a proton on the coordination site stabilizes Ti^3+^–peroxo species, thereby facilitating oxygen atom transfer.

Thus, the superior efficiency of TiO_2_-TBOT is attributed not only to morphological factors but also to its specific electronic and acidic structure, which promotes the formation and regeneration of active Ti–peroxo species, making this approach highly promising for industrial desulfurization applications (Zu et al., 2019).

### 3.5 Oxidation mechanisms in the presence of carboxylic acids and peroxyacids

**FIGURE 8:**
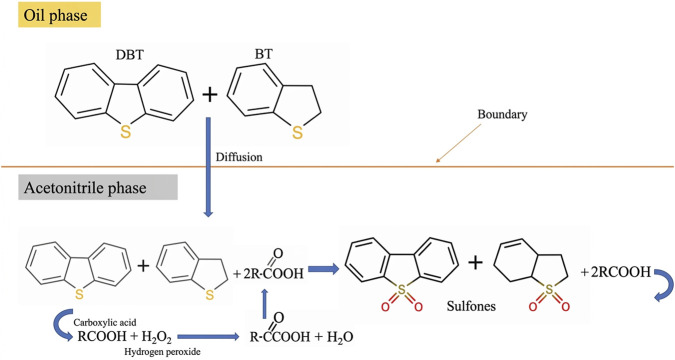
Reaction mechanism of oxidative desulfurization using *in situ* generated organic peroxy acids.

In the study (Ahmed et al., 2022) described a two-step mechanism for the oxidation of sulfur compounds in diesel fuel. In the first step, organic peracids (RCOOOH) are generated through the reaction of hydrogen peroxide with a dicarboxylic acid. The carboxyl group (–COOH) is readily converted into a peroxycarboxyl group under the action of H_2_O_2_. Subsequently, these *in situ* formed peracids oxidize divalent sulfur in thiophenes and their derivatives to sulfoxides and then to sulfones, without cleavage of the C–S bonds (Figure 8).

Sulfones are polar compounds with poor solubility in the hydrocarbon phase; therefore, their subsequent removal is typically carried out by extraction. The formation of peracid is critical for initiating the mechanism: without it, the reaction does not proceed or occurs with very low conversion. Thus, the key element of the mechanism is the generation of active oxygen species (peracids) capable of selectively oxidizing sulfur to sulfones under mild conditions (without metal catalysts and high temperatures). Thiophene structures exhibit high resistance to oxidation: under ILs–H_2_O_2_ conditions their conversion is limited, which is attributed to π-electron delocalization in the aromatic ring and the low reactivity of sulfur in this configuration. The role of the ionic liquid ([BMIM][BF_4_]) is not only to improve wetting of the carbon surface and increase the accessibility of active sites, but also to stabilize active oxygen species (Xu et al., 2020). This creates a favorable medium for selective and targeted oxidation of sulfur-containing fragments. As a result, the ILs–H_2_O_2_ system enables efficient and controlled oxidative transformation of organically bound sulfur in coal, predominantly yielding sulfones as intermediate or final products that can subsequently be removed.

**FIGURE 9:**
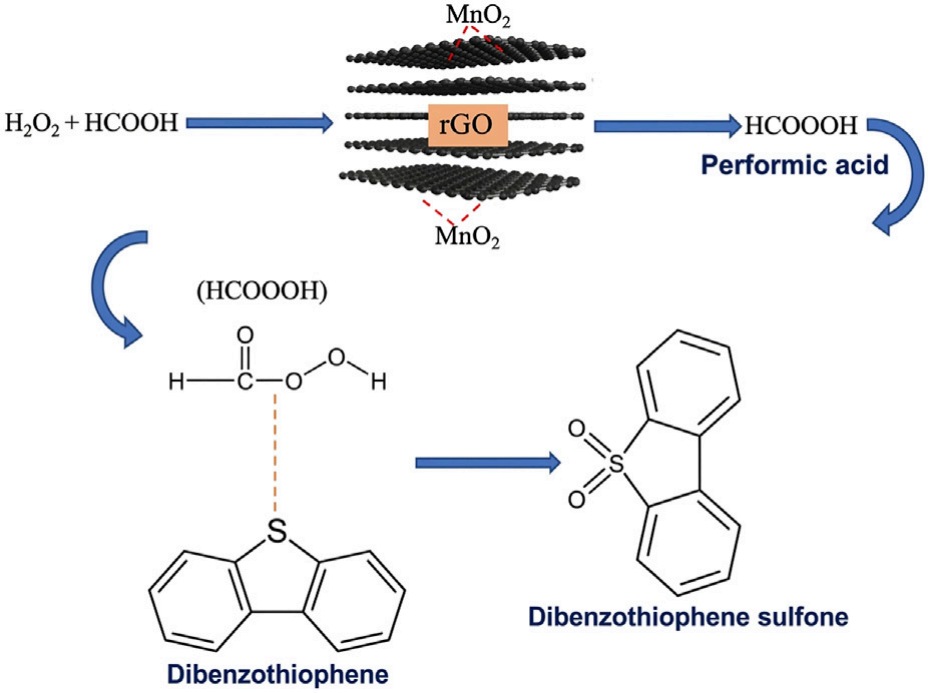
Proposed mechanism for oxidative desulfurization of dibenzothiophene using *in situ* generated performic acid (HCOOOH) in the presence of MnO_2_/MgO catalyst.

Oxidative desulfurization (ODS) of DBT proceeds with high efficiency in the presence of a MnO_2_/MrGO composite catalyst (Song et al., 2025)). As illustrated in the proposed mechanism, the reaction is initiated by MnO_2_, which catalyzes the heterolytic cleavage of the hydrogen peroxide molecule, leading to the formation of hydroxyl radicals (•OH)—powerful oxidizing agents. These radicals subsequently react with formic acid to produce performic acid (HCOOOH), a strong oxidant. It is this performic acid that directly oxidizes the organosulfur compound (DBT) to the corresponding sulfone (Figure 9).

MnO_2_ within the composite plays a key role, significantly enhancing the efficiency of peroxo-species formation and their subsequent participation in oxidation. In contrast, MrGO and GO, although possessing sorption properties, contribute much less to the overall activity of the system compared to MnO_2_. This is confirmed by the comparison of DBT conversion: 41% with GO, 53% with MrGO, and 80% when using MnO_2_/MrGO. Thus, MnO_2_ in the MnO_2_/MrGO composite catalyzes the formation of active oxidants and is the decisive component for the effective implementation of the ODS process.

Similarly, in the study *An experimental investigation on the oxidative desulfurization of a mineral lubricant base oil* by Ahmad Mortezaee, Mohammad Amin Sobati, Salman Movahedirad, and Shahrokh Shahhosseini (Mortezaee et al., 2021), a hydrogen peroxide–formic acid system was effectively employed for the oxidative desulfurization (ODS) of sulfur-containing compounds such as dibenzothiophene (DBT). The key oxidant in this process is performic acid (HCOOOH), which is generated *in situ* in the aqueous phase via the reaction between H_2_O_2_ and HCOOH (Equation 10):

$$\text{HCOOH}+H_{2}O_{2}\to \text{HCOOOH}+H_{2}O$$ (10)

The performic acid formed is unstable and can either decompose into formic acid, water, carbon dioxide, and oxygen (Reaction 4), or react with sulfur-containing compounds, oxidizing them to sulfoxides and sulfones (Equations 11, 12):

$$3\text{HCOOOH}\to 2\text{HCOOH}+H_{2}O+{\text{CO}}_{2}+O_{2}$$ (11)

$$2\text{HCOOOH}+\text{DBT}\to 2\text{HCOOH}+{\text{DBTO}}_{2}$$ (12)

Thus, in this system HCOOH acts as a catalyst, whereas H_2_O_2_ serves as the primary consumed oxidant. After mixing the aqueous phase (containing HCOOH, H_2_O_2_, HCOOOH, and water) with the organic phase (petroleum fraction), performic acid transfers into the hydrocarbon phase and oxidizes the sulfur-containing compounds dissolved therein.

### 3.6 Catalytic oxidation pathways mediated by polyoxometalates in ODS

The synthesis of Brønsted acidic ionic liquids (ILs) for DBT oxidation based on phosphotungstic acid and various morpholine derivatives was reported in (Eseva et al., 2020) (Figure 9).

**FIGURE 10:**
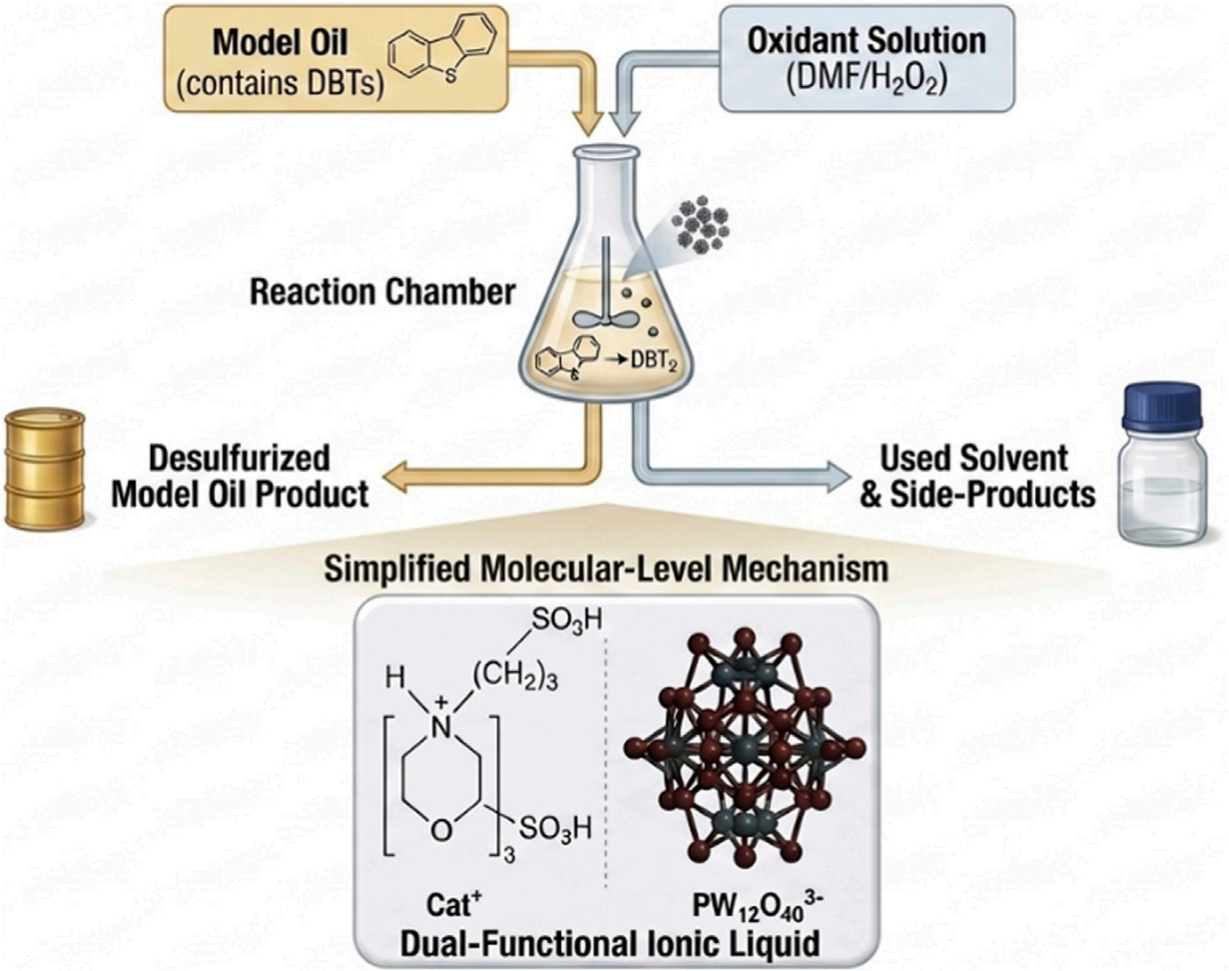
Mechanism of DBT oxidation in the presence of [C3SO3Hnhm]_3_PW_12_O_40_.

It was found that the series of samples containing the–SO_3_H functional group and exhibiting strong Brønsted acidity showed superior catalytic activity (Brillas and Martínez-Huitle, 2015; Zu et al., 2019). In particular, in the case of [C3SO3Hnhm]_3_PW_12_O_40_, the DBT content was reduced by 99.4%. Although heteropolyacids (HPAs) can actively contribute to this reaction through the formation of peroxo complexes with H_2_O_2_, the role of Brønsted acid sites should not be underestimated, as hydrogen bonding between the sulfur atom and the sulfonic group renders the substrate more susceptible to oxidation (Figure 10).

The role of Brønsted acid sites in desulfurization is demonstrated using the example of the interaction between thiophene and an ionic liquid (IL) containing an acidic hydrogen atom (Chen et al., 2025; Gorbunov et al., 2023; Zhang et al., 2012). In the IR spectrum of the [Hnmp]BF_4_–thiophene adduct, the appearance of a new absorption band at 735 cm^-1^ and the weakening of the band at 713 cm^-1^ are observed, indicating a distortion of the thiophene structure. The intensity of this band correlates with the catalytic activity of the various derivatives.

^1^H NMR data show a downfield shift of the acidic proton signal upon the addition of thiophene to [Hnmp]BF_4_, providing direct evidence for the formation of hydrogen bonds between the ionic liquid and the substrate.

A key feature of this catalytic system is the implementation of a dual activation mechanism of the substrate. According to spectroscopic studies and cyclic voltammetry data, Brønsted acidic ionic liquids form hydrogen bonds with sulfur-containing aromatic compounds. This interaction leads to a distortion of the planar structure of thiophene and a decrease in its aromaticity, which significantly facilitates the oxidation process.

Experimental data indicate that the activation energy of benzothiophene oxidation in the presence of [Hnmp]BF_4_ is less than 40 kJ mol^-1^, whereas in the [Bmim]BF_4_ system it reaches 76.8 kJ mol^-1^. Such a substantial decrease in the energy barrier is attributed to the synergistic effect of hydrogen bonding and the catalytic function of tungstate.

The support material also plays an important role in oxidation-based removal of heteroatom-containing substrates in the presence of supported ionic liquids (Ali-Zade et al., 2020; Bodaghifard et al., 2023). In study (Jiang et al., 2020b), silica impregnated with the ionic liquid derivative [Omim][HSO_4_] (1-octyl-3-methylimidazolium hydrogensulfate) was tested as a catalyst for the oxidation of DBT, BT, and 4,6-DMDBT in a hydrocarbon medium with hydrogen peroxide. According to the proposed mechanism, substrate molecules are adsorbed on the catalyst surface and subsequently diffuse into the pore system. There, due to π–π interactions between the imidazolium ring and the sulfur atom, the intermediate is extracted into the ionic liquid phase, where oxidation proceeds to the corresponding derivatives via hydroxyl radicals generated from the interaction between the IL and the oxidant. This system was also found to be effective for the removal of sulfur compounds from a diesel fraction after preliminary hydrodesulfurization: within 50 min at 50 °C, the sulfur content decreased from 746 to 180 ppm.

A series of PMo/ILSiO_2_ samples, in which an ionic liquid derivative based on [Bmim]_3_PMo_12_O_40_ (1-butyl-3-methylimidazolium bromide) and phosphomolybdic acid (PMA) was covalently anchored onto the silica surface, was reported in (Zhang et al., 2018). The proposed scheme illustrates the oxidation mechanism of dibenzothiophene (DBT) to sulfone (DBTO_2_) using the polyoxometalate catalyst PMo/ILSiO_2_, in which the Keggin-type phosphomolybdate anion is covalently immobilized on the silica surface through the ionic liquid [Bmim]_3_PMo_12_O_40_.

**FIGURE 11:**
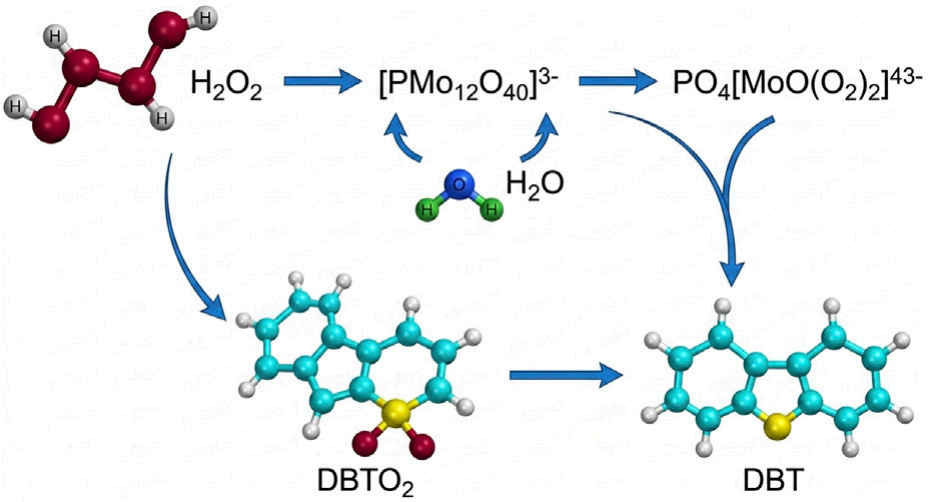
Proposed peroxo-molybdate pathway for DBT-to-DBTO_2_ oxidation with H_2_O_2_ over the PMo/ILSiO_2_ catalyst.

The interaction of the heteropolyanion with H_2_O_2_ leads to the formation of highly active peroxo-molybdenum complexes capable of efficiently transferring oxygen atoms. DBT molecules are adsorbed on the active sites through coordination of the sulfur atom with the electrophilic peroxo groups and via π–π interactions between the aromatic core and the imidazolium ring of the IL. The oxidation proceeds in two steps—first to sulfoxide (DBTO) and then to sulfone (DBTO_2_)—while maintaining the integrity of the C–S bond (Figure 11).

The mechanism of DBT oxidative desulfurization was described using hybrid catalysts composed of an organic cationic moiety, [H_2_TPP]^2+^, and the inorganic anion [BVW_11_O_40_]^7-^ (Hassan et al., 2022).

**FIGURE 12:**
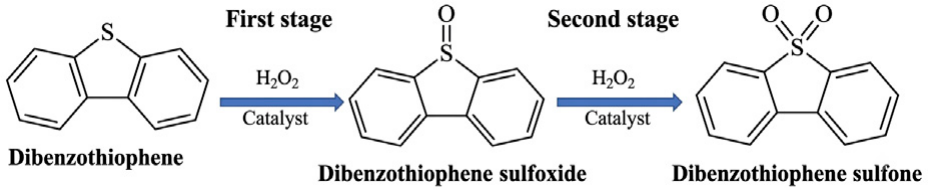
Sequential oxidation of DBT to DBTO and DBTO_2_ with H_2_O_2_ over the [H_2_TPP]^2+^[BVW_11_O_40_]^7-^ hybrid catalyst via peroxo-tungsten intermediates.

The terminal oxygen atoms act as the active sites, which, in the presence of H_2_O_2_, are transformed into tungsten peroxo-complexes capable of transferring oxygen atoms to the substrate. The reaction proceeds through the sequential formation of sulfoxide (DBTO) and its further oxidation to sulfone (DBTO_2_), while the peroxo groups are regenerated by H_2_O_2_, ensuring a closed catalytic cycle (Figure 12).

**FIGURE 13:**
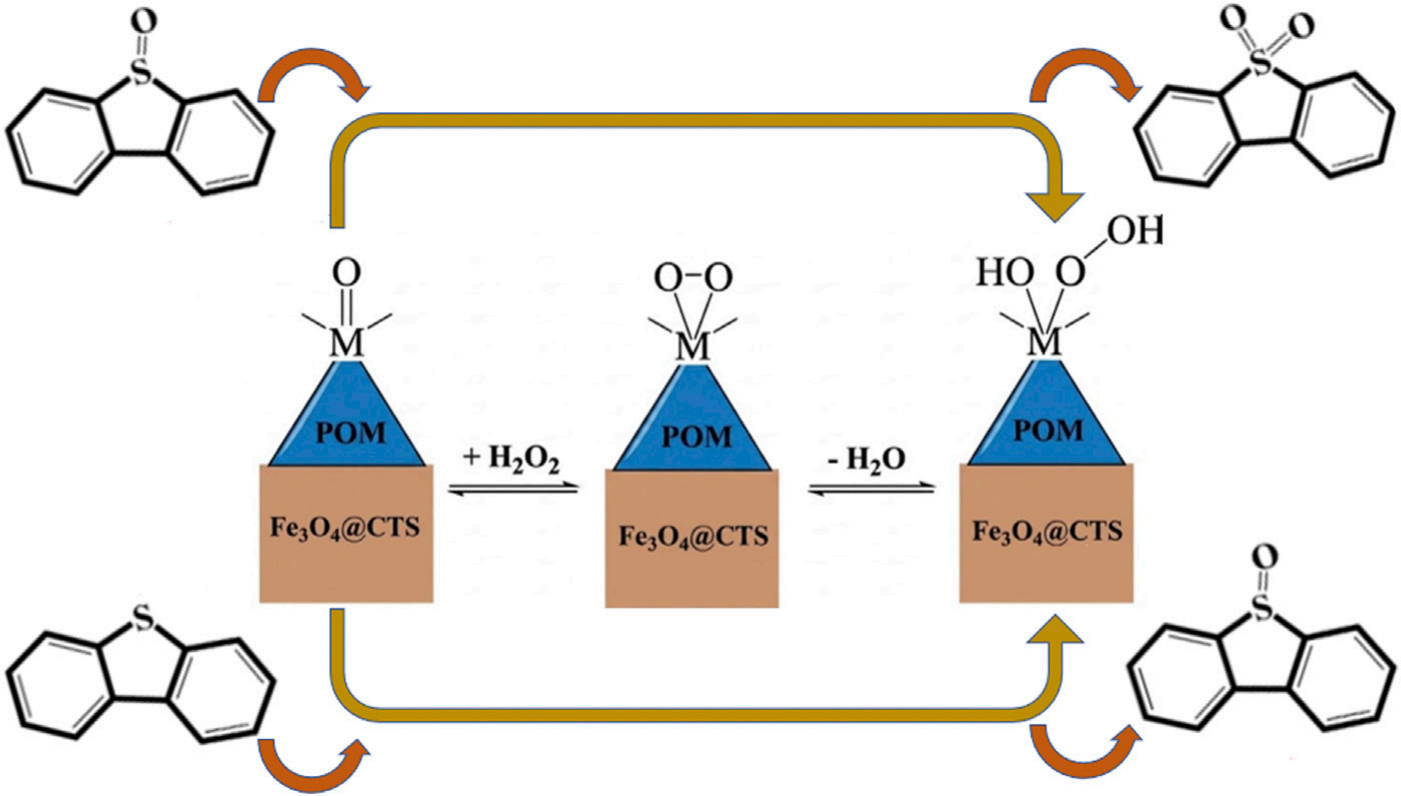
Two-step oxidation of DBT over POM/Fe_3_O_4_@CTS via peroxo- and hydroperoxo-metal intermediates regenerated by H_2_O_2_.

In the POM/Fe_3_O_4_@CTS system (Gooneh-Farahani and Anbia, 2025), oxidative desulfurization also proceeds via the sequential formation and regeneration of metal peroxo species. Under the action of H_2_O_2_, the terminal oxo groups of the metal in the POM structure are transformed into active peroxo complexes (M–O–O), which initiate the two-step oxidation of organosulfur compounds: first to sulfoxide and then to sulfone (Figure 13).

During the reaction, the peroxo complexes are reduced back to the parent POM, which is subsequently re-oxidized by hydrogen peroxide, thus closing the catalytic cycle. The presence of alkyl substituents in aromatic sulfur-containing compounds increases the electron density on the sulfur atom, facilitating electrophilic oxygen transfer and accelerating the process.

## 4 Conclusion

The efficiency of H_2_O_2_-based ODS hinges upon the effective activation of H_2_O_2_ to yield reactive oxygen species (ROS), including hydroxyl radicals (•OH), hydroperoxyl radicals (HO_2_•), and superoxide anions (O_2_•^-^), which selectively oxidize organosulfur compounds to their polar sulfoxide and sulfone derivatives. This mechanistic foundation has driven the development of a diverse range of catalytic systems, encompassing polyoxometalates, metal-organic frameworks, deep eutectic solvents, ionic liquids, transition metal oxides, and metal-free catalysts. Many of these systems have demonstrated sulfur removal efficiencies exceeding 90%, with some approaching complete desulfurization in both model and real fuel matrices (Anisimov et al., 2003; Armandsefat et al., 2024; Jiang et al., 2020a; Messele et al., 2019; Meyerstein, 2021; Torres-García et al., 2011).

One of the main challenges in H_2_O_2_-assisted oxidative desulfurization (ODS) is the coexistence of multiple reactive oxygen species (ROS), making it difficult to identify the dominant oxidant under specific conditions. Catalyst efficiency strongly depends on surface structure, defects, porosity, and metal coordination, especially for bulky sulfur compounds like 4,6-DMDBT. The reaction typically occurs at the oil–water interface, yet the exact site of H_2_O_2_ activation and transport pathways remain unclear. Diffusion limitations may hinder the intrinsic kinetics of the process. Catalyst deactivation, such as sulfone poisoning or pore blockage, along with efficient product removal, is a major concern for long-term operation. Non-selective decomposition of H_2_O_2_ significantly reduces efficiency, requiring strategies to suppress side reactions. Additionally, solvent properties can influence ROS generation and catalyst–oxidant–substrate interactions and should be carefully considered during process design.

Future studies should focus on scaling up stable and recyclable H_2_O_2_-ODS systems for real fuel applications with complex compositions. Deeper understanding of ROS generation, especially non-radical pathways, will support the development of more selective and durable catalysts. Emphasizing interfacial engineering, mass transport modeling, and metal-free or earth-abundant catalysts like hexagonal boron nitride will promote sustainable fuel production. Overall, H_2_O_2_-ODS represents a promising route to ultra-low sulfur fuels, reducing SO_x_ emissions and improving air quality.
